# AMP-activated protein kinase promotes epithelial-mesenchymal transition in cancer cells through Twist1 upregulation

**DOI:** 10.1242/jcs.208314

**Published:** 2018-07-26

**Authors:** Meera Saxena, Sai A. Balaji, Neha Deshpande, Santhalakshmi Ranganathan, Divya Mohan Pillai, Sravanth Kumar Hindupur, Annapoorni Rangarajan

**Affiliations:** Department of Molecular Reproduction, Development and Genetics, Indian Institute of Science, Bangalore 560012, Karnataka, India

**Keywords:** AMPK signaling, EMT, Twist

## Abstract

The developmental programme of epithelial-mesenchymal transition (EMT), involving loss of epithelial and acquisition of mesenchymal properties, plays an important role in the invasion-metastasis cascade of cancer cells. In the present study, we show that activation of AMP-activated protein kinase (AMPK) using A769662 led to a concomitant induction of EMT in multiple cancer cell types, as observed by enhanced expression of mesenchymal markers, decrease in epithelial markers, and increase in migration and invasion. In contrast, inhibition or depletion of AMPK led to a reversal of EMT. Importantly, AMPK activity was found to be necessary for the induction of EMT by physiological cues such as hypoxia and TGFβ treatment. Furthermore, AMPK activation increased the expression and nuclear localization of Twist1, an EMT transcription factor. Depletion of Twist1 impaired AMPK-induced EMT phenotypes, suggesting that AMPK might mediate its effects on EMT, at least in part, through Twist1 upregulation. Inhibition or depletion of AMPK also attenuated metastasis. Thus, our data underscore a central role for AMPK in the induction of EMT and in metastasis, suggesting that strategies targeting AMPK might provide novel approaches to curb cancer spread.

## Abstract (precis)

**Highlighted Article:** Pharmacological and physiological activation of AMPK promotes epithelial-mesenchymal transition in cancer cells through Twist1 upregulation and its increased nuclear localization.

## INTRODUCTION

Metastasis, the spread of tumor cells from a primary site to a distant organ, is accountable for more than 90% of cancer-related mortality (Chaffer and Weinberg, 2011). This process involves local invasion of cells from a primary tumor into the surrounding tissue, intravasation into the nearby microvasculature, migration through the blood and lymphatic vessels, extravasation at a distant organ site, and their proliferation to form a secondary macroscopic tumor (Chaffer and Weinberg, 2011). It is believed that the evolutionarily conserved developmental program of epithelial-mesenchymal transition (EMT) bestows cancer cells with properties that enable them to initiate the invasion-metastasis cascade (Yang et al., 2006). Indeed, EMT is considered to be an important contributor in the invasion and metastasis of several types of cancer (Thiery et al., 2009).

During EMT, epithelial cells shed their intercellular contacts, cell-extracellular matrix interactions and apico-basal polarity, and attain more mesenchymal properties such as increased expression of mesenchymal markers, motility and invasive capabilities (Polyak and Weinberg, 2009). EMT can be stimulated by a plethora of cues such as hypoxia, signaling through TGFβ, receptor tyrosine kinases, Notch and Wnt (Polyak and Weinberg, 2009). In response to these cues, several transcription factors such as Twist1, Snail (also known as SNAI1), Slug (also known as SNAI2), FOXC2, Zeb1 and Zeb2 (Polyak and Weinberg, 2009) are upregulated, which serve as important downstream mediators of EMT. Of these, Twist1 is considered as a master regulator of EMT (Yang et al., 2004). Several mechanisms of regulation of EMT have been elucidated in recent years (Polyak and Weinberg, 2009). However, a discreet hierarchy or a central orchestrator linking these various mechanisms in the EMT interactome has yet to be elaborated.

AMP-activated protein kinase (AMPK) is an evolutionarily conserved stress-sensing kinase that becomes activated under a variety of stresses, including hypoxia, ischemia and nutrient deprivation, leading to a change in the ATP:AMP ratio (Steinberg and Kemp, 2009). It is a heterotrimeric serine/threonine protein kinase consisting of a catalytic α subunit (α1 or α2), a scaffolding β subunit (β1 or β2) and a nucleotide-binding γ subunit (γ1, γ2 or γ3) (Hardie et al., 2012). Besides its pertinent role in the regulation of energy homeostasis, AMPK has been implicated in several physiological processes including cell growth (Xiang et al., 2004), cell division (Jones et al., 2005), maintenance of epithelial cell polarity (Zhang et al., 2006) and autophagy (Liang et al., 2007). Apart from these, AMPK activity has also been associated with cell migration in normal physiology (Nakano et al., 2010). For instance, AMPK activation was shown to be important for migration of human umbilical vein endothelial cells (HUVECs) (Nagata et al., 2003) and transendothelial lymphocyte migration (Martinelli et al., 2009). In another study, inhibition of AMPK was shown to suppress TGFβ-mediated apoptosis and EMT of normal murine hepatocytes (Wang et al., 2010). A few reports have also associated AMPK signaling with cancer cell migration and invasion (Chen et al., 2011; Chiu et al., 2009; Kim et al., 2011). Another recent study showed that indirect activation of AMPK by mitochondrial dysfunction induced EMT in lung cancer cells (He et al., 2016). In contrast, some studies have reported an inhibitory role for AMPK in EMT of breast, prostate and lung cancer cells, mainly using 5-aminoimidazole-4-carboxamide ribonucleotide (AICAR) and metformin as pharmacological activators of AMPK (Banerjee et al., 2016; Chou et al., 2014; Cufí et al., 2010; Han et al., 2015; Lin et al., 2015; Qu et al., 2014). Thus, there are conflicting reports about the role of AMPK signaling in EMT and cancer metastasis, suggesting that AMPK activation might have cell-type- and context-specific effects. This might, in turn, depend on the upstream activators of AMPK used, some of which could additionally have AMPK-independent effects. This incongruity needs to be resolved before AMPK-targeted therapeutics are widely used for cancer treatment.

In light of these conflicting reports, we investigated the role of AMPK in regulating EMT in multiple cancer types. In the present study, we show that AMPK activation with a direct, allosteric activator A769662 suffices to induce a functional EMT in multiple cancer cells, whereas its inhibition or depletion leads to reversal of EMT. Importantly, we show that AMPK is required for EMT induction by upstream stimuli relevant to cancer progression, such as hypoxia and TGFβ. Furthermore, we show that AMPK mediates its effects, at least in part, through upregulation of Twist1, a major regulator of EMT, and increasing its nuclear localization.

## RESULTS

To explore a possible role for AMPK in EMT, we initiated our study by investigating the effects of AMPK activation and inhibition on the expression of markers associated with EMT in a variety of cancer cell lines including breast cancer cell lines MCF7, T47D, BT474 and MDA-MB-231, melanoma cell line MDA-MB-435S and lung adenocarcinoma cell line A549. First, we determined the endogenous expression of epithelial marker E-cadherin (E-cad) and mesenchymal markers vimentin (Vim) and N-cadherin (N-cad) in these cell lines (Fig. S1A). The cancer cell lines BT474, MCF7 and T47D exhibiting an E-cad^+^ and Vim^−^ phenotype represent epithelial cell types. A549 exhibited an E-cad^low^ and Vim^low^ phenotype, reminiscent of a partially epithelial-mesenchymal-transitioned cell line, whereas MDA-MB-231 and MDA-MB-435S cells exhibited an E-cad^−^ and Vim^high^ phenotype, representing mesenchymal cell types. The pattern of expression of these markers matched that reported in the literature (Lombaerts et al., 2006).

**Fig. 1.**
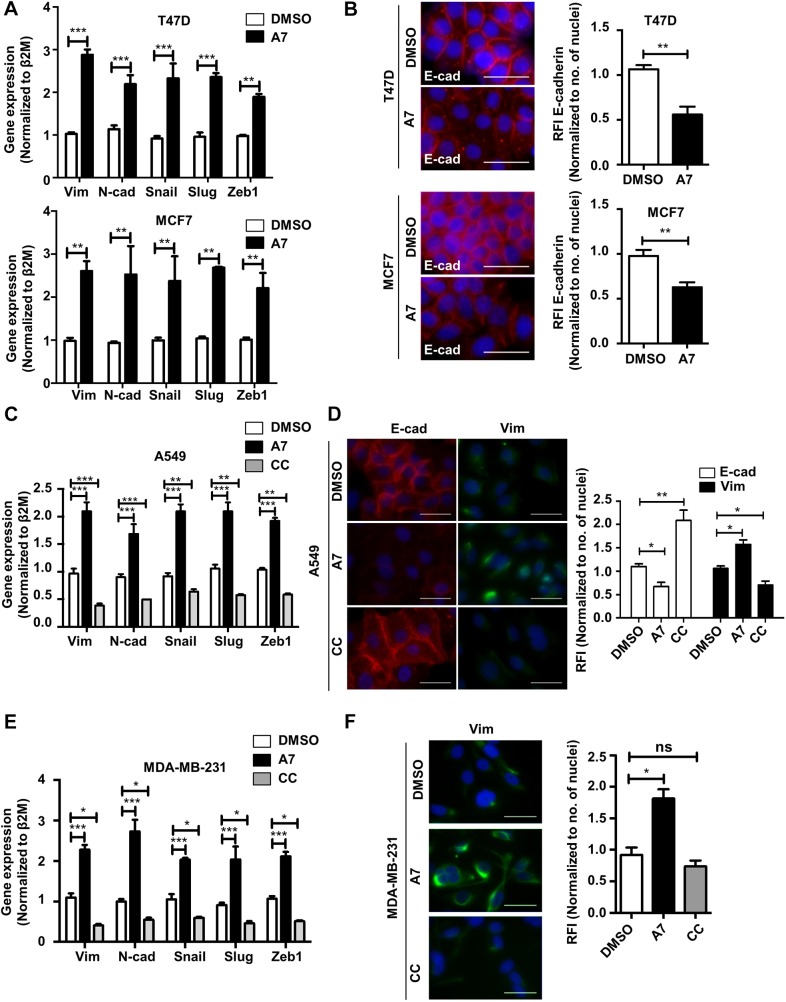
**AMPK activity modulates EMT marker expression.** (A-F) Cancer cell lines T47D, MCF7 (A,B), A549 (C,D) and MDA-MB-231 (E,F) were cultured in the presence of 100 μM AMPK activator (A769662), 10 μM AMPK inhibitor (Compound C) or DMSO (vehicle control) as specified for 48 h. Thereafter, cells were harvested and subjected to qRT-PCR analysis for the specified transcripts. Graphs in panels A, C and E represent fold change in gene expression normalized to β2M; error bars represent s.e.m., *n*=3. Parallel dishes were fixed using 4% paraformaldehyde to undertake immunocytochemistry analysis for the specified proteins (B,D,F). Photomicrographs show representative fluorescent images taken using an Olympus 1X71 microscope at 20× magnification and processed using ImageJ software. E-cad is stained in red; Vim is stained in green; the nucleus is stained in blue (Hoechst 33342). Graphs in panels B, D and F represent relative fluorescence intensity measurements normalized to number of nuclei. Error bars represent s.e.m., *n*=3. Scale bars: 20 μm. *P<0.05; **P<0.01; ***P<0.001; ns, not significant. A7, A769662; CC, Compound C.

To begin our study, we investigated whether AMPK activation would promote the induction of EMT in epithelial-type cancer cell lines. Most previous studies have used AICAR or metformin for activating AMPK; however, these have additional AMPK-independent effects as well (Foretz et al., 2010; Kalender et al., 2010). To overcome this problem, we used A769662 (Cool et al., 2006), which directly activates the heterotrimeric form of AMPK both allosterically and by inhibiting its dephosphorylation (Sanders et al., 2007). We confirmed treatment with A769662 by performing immunoblot analyses to detect the levels of phosphorylated acetyl-CoA carboxylase (ACC), a bona fide substrate of AMPK (Witters et al., 1991). An increase in the phosphorylation of ACC is indicative of AMPK activation (Fig. S1B). Treatment of T47D and MCF7 cells with A769662 led to an increase in the transcript levels of mesenchymal markers N-cad and Vim and EMT transcription factors Snai1, Slug and Zeb1 (Fig. 1A). Moreover, since decrease in the protein levels of epithelial markers and disruption of adherens junctions are hallmarks of EMT (Huang et al., 2012), we also investigated the effects of AMPK activation on the protein levels of the epithelial markers epithelial cadherin (E-cad; also known as CDH1) and zona occludens protein 1 (ZO-1, also known as TJP1). Immunocytochemical analysis revealed a significant reduction in E-cad levels upon AMPK activation for 48 h in these cell lines (Fig. 1B). Reduced expression of E-cad and ZO-1 was also detected using an immunoblotting approach (Fig. S1B). Similar results were obtained with AMPK activation in BT474 cells (Fig. S1C,D,E). However, in spite of the changes at transcript levels (Fig. 1A and Fig. S1C), AMPK activation for 48 h did not lead to the detection of the mesenchymal markers Vim and N-cad at protein levels in these epithelial cell types (data not shown). Thus, this set of data revealed that AMPK activation in the epithelial cancer cells suffices to bring about a partial EMT involving the downmodulation of epithelial markers.

Next, we investigated the effects of AMPK activation and inhibition on cancer cells that had already undergone EMT to various extents, such as A549, MDA-MB-231 and MDA-MB-435S cells. Activation of AMPK in the partially epithelial-mesenchymal-transitioned A549 cells, bearing an E-cad^low^ and Vim^low^ phenotype, led to an increase in the mRNA levels of mesenchymal markers and EMT transcription factors (Fig. 1C). Immunofluorescence analysis also revealed a reduction in E-cad and increase in Vim protein expression (Fig. 1D). Reduction in the expression of the epithelial markers E-cad and ZO-1, and increase in the expression of the mesenchymal markers Vim and N-cad, was also detected using immunoblotting (Fig. S1F). Thus, AMPK activation further promoted the EMT phenotype of A549 cells. In contrast, inhibition of AMPK using 10 μM Compound C, a potent, reversible, and ATP*-*competitive inhibitor of AMPK (Zhou et al., 2001), led to a reduction in Vim and N-cad expression at both mRNA and protein levels, and an increase in E-cad and ZO-1 protein levels (Fig. 1C,D and Fig. S1F).

In MDA-MB-231 cells bearing an E-cad^−^ and Vim^high^ phenotype, AMPK activation led to a further increase in the mRNA expression of mesenchymal markers Vim and N-cad and EMT transcription factors Snai1, Slug and Zeb1, whereas AMPK inhibition led to a reduction in their transcript levels (Fig. 1E). At protein levels, we observed a significant increase in Vim expression upon AMPK activation and a moderate decrease upon AMPK inhibition (Fig. 1F and Fig. S1G). As reported previously (Sommers et al., 1991), we failed to detect the expression of E-cad in MDA-MB-231 cells (Fig. S1A), and AMPK inhibition also did not lead to its re-expression in these cells (data not shown). However, AMPK activation led to a decrease in the expression of ZO-1, whereas its inhibition led to increased ZO-1 in MDA-MB-231 cells (Fig. S1G). Similar results were obtained with another mesenchymal cell line, MDA-MB-435S (Fig. S1H).

Because pharmacological inhibition of AMPK (with Compound C) might have off-target effects (Liu et al., 2014), we additionally undertook RNAi-mediated depletion of AMPK. To do so, we used BT474 and A549 cells stably expressing AMPKα2 shRNA under constitutive and inducible promoters, respectively, each showing ∼50% and ∼70% knockdown of AMPK (Fig. S1I and J). Depletion of AMPKα2 in the epithelial BT474 cells led to a further increase in the expression of epithelial markers E-cad and ZO-1 (Fig. S1I), although we failed to see any expression of Vim (data not shown). In the partially epithelial-mesenchymal-transitioned A549 cells, AMPK knockdown led to an increase in the expression of E-cad and ZO-1 while reducing the levels of Vim (Fig. S1J; clone #1). Immunofluorescence analysis further revealed an increase in E-cad expression and a decrease in Vim expression upon AMPK depletion in these cells (Fig. S1K). Similar results were obtained in A549 cells where AMPK was stably knocked down using another shRNA oligo sequence (Fig. S1L; clone #4). Similarly, mesenchymal MDA-MB-231 cells that stably express AMPKα2 shRNA (Hindupur et al., 2014) and showed ∼80% knockdown (Fig. S1M) revealed a reduction in the levels of Vim and N-cad (Fig. S1M,N). These results were validated further using an additional, inducible shRNA targeting AMPKα2 (Fig. S1O; clone #4) in MDA-MB-231 cells. Similar results were obtained upon AMPK knockdown in yet another mesenchymal cell type, MDA-MB-435S (Fig. S1P). Thus, these data revealed that in cancer cells that have undergone EMT to varying extent, AMPK activation further promoted, whereas its inactivation or depletion reversed the morphological changes associated with EMT, suggesting that AMPK plays a key role in regulating the process of EMT.

**Fig. 2.**
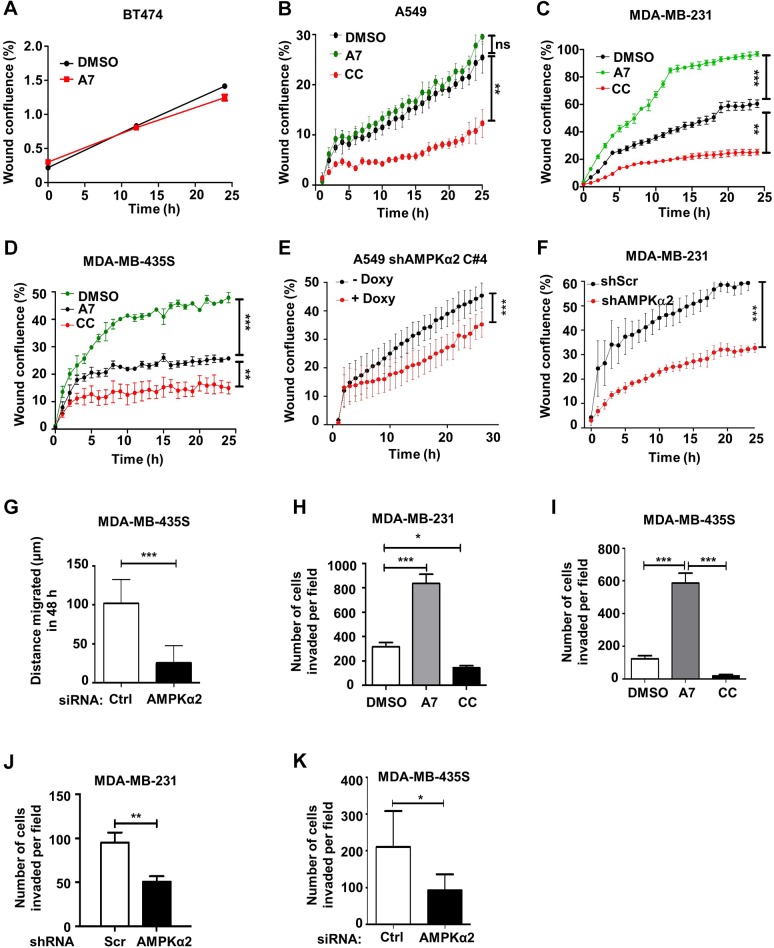
**AMPK induces a functional EMT.** (A) Scratch assay was performed with BT474 cells after treatment with 100 µM AMPK activator (A769662; A7) or DMSO (vehicle control). The graph represents time kinetics of wound confluence percentage, calculated by IncuCyte ZOOM software. (B) Scratch assay was performed with A549 cells after treatment with 100 µM AMPK activator (A769662), 10 µM AMPK inhibitor (Compound C) or DMSO (vehicle control). The graph represents time kinetics of wound confluence percentage, calculated by IncuCyte ZOOM software. Error bars represent s.e.m., *n*=2. (C,D) Scratch assay was performed with MDA-MB-231 (C) and MDA-MB-435S (D) cells after treatment with 100 µM AMPK activator (A769662), 10 µM AMPK inhibitor (Compound C) or DMSO (vehicle control). Graphs represent time kinetics of wound confluence percentage, calculated by IncuCyte ZOOM software. Error bars represent s.e.m., *n*=3 and 2, respectively. (E) Scratch assay was performed with A549 cells stably expressing inducible shRNA against AMPKα2 (clone #4) cultured with and without doxycycline. The graph represents time kinetics of wound confluence percentage, calculated by IncuCyte ZOOM software. Error bars represent s.e.m., *n*=2. (F) Scratch assay was performed with MDA-MB-231 cells stably expressing scrambled shRNA or AMPKα2 shRNA. The graph represents time kinetics of wound confluence percentage, calculated by IncuCyte ZOOM software. Error bars represent s.e.m., *n*=2. (G) Scratch assay was performed with MDA-MB-435S cells transfected with control siRNA or AMPKα2 siRNA. Graphs represent the distance migrated between the cell edges by 48 h, calculated by ImageJ software. Error bars represent s.e.m., *n*=3. (H,I) Boyden chamber invasion assay was performed for 24 h with MDA-MB-231 (H) and MDA-MB-435S cells (I) treated with 100 µM AMPK activator (A769662), 10 µM AMPK inhibitor (Compound C) or DMSO (vehicle control). Graphs represent number of cells invaded per field (9-10 fields were counted per experiment). Error bars represent s.e.m., *n*=3. (J,K) Boyden chamber invasion assay was performed for 24 h with MDA-MB-231 cells stably expressing scrambled shRNA or AMPKα2 shRNA; *n*=2 (J) or MDA-MB-435S cells transfected with control siRNA or AMPKα2 siRNA; *n*=3 (K). Graphs represent number of cells invaded per field (9-10 fields were counted per experiment). Error bars represent s.e.m. For all scratch/migration assay experiments above, cells were pretreated with 10 µg/ml Mitomycin C for 2 h before the scratch/wound was made. For all migration assay experiments performed using IncuCyte ZOOM, 10× magnification (wide mode) was used. *P<0.05; **P<0.01; ***P<0.001. A7, A769662; CC, Compound C; Ctrl, control; Scr, scrambled; sh, shRNA.

Attainment of motility and invasive capabilities are hallmark features of a functional EMT (Polyak and Weinberg, 2009). To assess whether AMPK can modulate the motility and migration of cancer cells, we undertook a scratch assay with BT474, A549, MDA-MB-231 and MDA-MB-435S cells treated with either AMPK activator A769662 or its inhibitor Compound C. In keeping with a failure to induce mesenchymal marker expression in epithelial cell lines, AMPK activation failed to alter migration of BT474 cells (Fig. 2A and Fig. S2A). In the partially epithelial-mesenchymal-transitioned A549 cells, A769662 treatment led to a marginal increase in migration whereas Compound C treatment resulted in a significant decrease in the motility (Fig. 2B and Fig. S2B). Interestingly, mesenchymal MDA-MB-231 and MDA-MB-435S cells showed a significant increase in migration when treated with A769662 and a significant inhibition of motility with Compound C treatment (Fig. 2C, Fig. S2C, Fig. 2D and Fig. S2D). To further confirm these data, we additionally undertook yet another cell migration assay with MDA-MB-435S cells involving quantitative real-time impedance measurement using an ECIS (electric cell-substrate impedance sensing)-based technique (Hong et al., 2011a). In corroboration with the scratch assay results, we found that although A769662 treatment led to a marked increase in migration of MDA-MB-435S cells (as revealed by an increase in impedance), Compound C treatment led to a decrease in migration (as revealed by a decrease in impedance) compared with the control cells (Fig. S2E). Further, to confirm the results with Compound C, we employed RNAi-mediated knockdown of AMPKα2. Consistent with pharmacological inhibition of AMPK, A549, MDA-MB-231 and MDA-MB-435S cells stably expressing AMPKα2 shRNA or transfected with siRNA targeting AMPKα2 also showed decreased migration compared with scrambled shRNA control cells (Fig. 2E, Fig. S2F, Fig. 2F, Fig. S2G, Fig. 2G and Fig. S2H). Knockdown of AMPK failed to change the migration in BT474 cells (data not shown). The effect of AMPK inhibition/knockdown on the migratory properties of mesenchymal cells (Fig. 2F, Fig. S2G, Fig. 2G and Fig. S2H) was more dramatic compared with the partially epithelial-mesenchymal-transitioned A549 cells (Fig. 2E and Fig. S2F).

To determine the effects of AMPK activation and inhibition on the invasive potential of cancer cells, we performed *in vitro* Boyden chamber invasion assays. Since mesenchymal cells showed maximal effect on AMPK modulation, we undertook invasion assays with these cell types. Compared with vehicle-treated control cells, we found that whereas A769662 treatment caused a significant increase in the invasiveness of MDA-MB-231 and MDA-MB-435S cells, Compound C treatment decreased their invasive potential (Fig. 2H,I). Furthermore, knockdown of AMPKα2 in MDA-MB-231 and MDA-MB-435S cells also led to a marked decrease in their invasive potential compared with control cells (Fig. 2J, Fig. S2I and Fig. 2K). These results collectively demonstrated that whereas AMPK activation increased the migratory and invasive capability of cancer cells, thereby bringing about a functional EMT, its inhibition or knockdown reversed EMT, suggesting that AMPK plays a critical role in the morphogenetic processes involved with EMT.

**Fig. 3.**
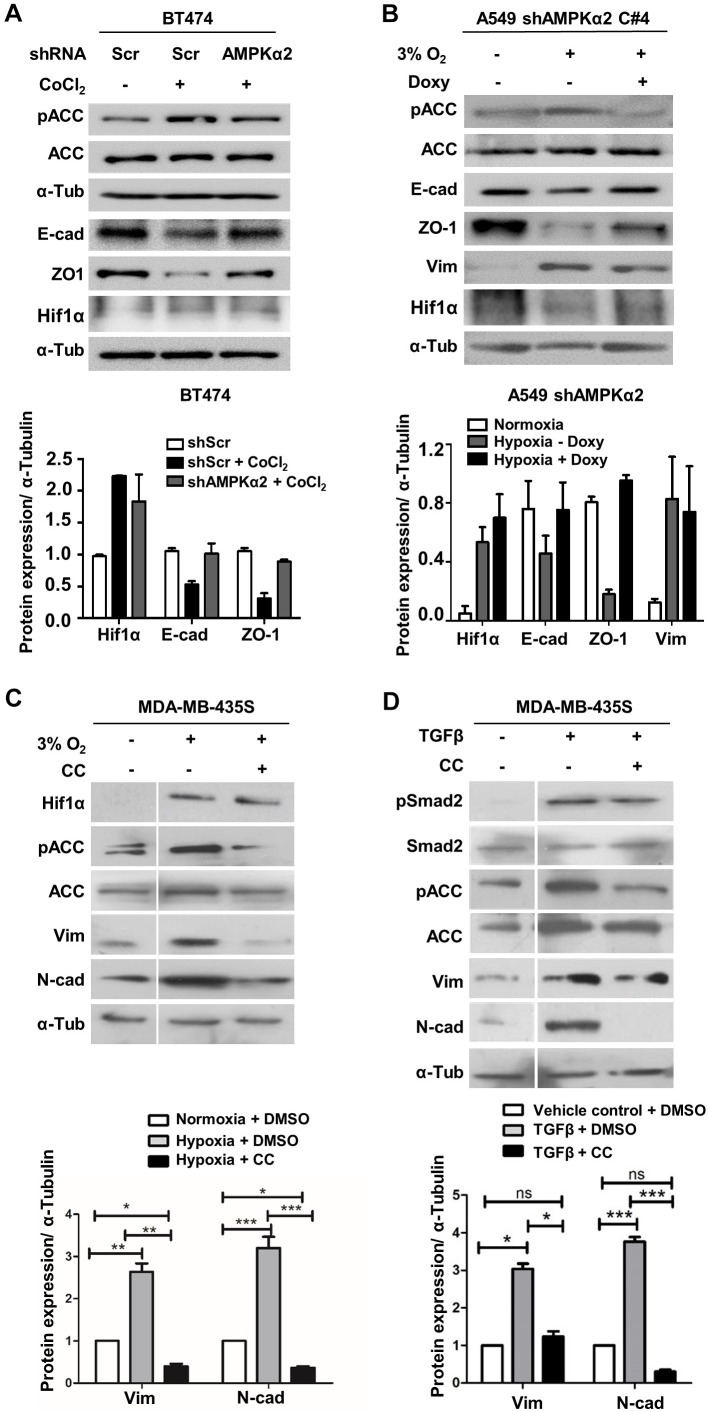
**AMPK is necessary for EMT induction by different upstream stimuli.** (A) BT474 cells stably expressing scrambled shRNA or AMPKα2 shRNA were cultured in the presence of 150 µM CoCl_2_ for 48 h. Thereafter, cells were harvested and immunoblot analysis was undertaken. The graph represents densitometric quantification of the specified proteins normalized to α-tubulin; error bars represent s.e.m.; *n*=2. (B) A549 cells stably expressing inducible shRNA against AMPKα2 (clone #4) were cultured with and without doxycycline in ambient oxygen (20% O_2_) or in hypoxic (3% O_2_) conditions in a tri-gas incubator for 48 h. Thereafter, cells were harvested and subjected to immunoblot analysis. The graph represents densitometric quantification of the specified proteins normalized to α-tubulin; error bars represent s.e.m.; *n*=2. (C) MDA-MB-435S cells were cultured in ambient oxygen (20% O_2_) or in hypoxic (3% O_2_) conditions in a tri-gas incubator in the presence of 10 µM AMPK inhibitor (Compound C) for 24 h. Thereafter, cells were harvested and subjected to immunoblot analysis. The graph represents densitometric quantification of the specified proteins normalized to α-tubulin; error bars represent s.e.m.; *n*=4. (D) MDA-MB-435S cells were cultured with 5 ng/ml TGFβ in the presence or absence of 10 µM AMPK inhibitor (Compound C) for 24 h. Thereafter, immunoblot analysis was undertaken for the specified proteins. The graph represents densitometric quantification of the specified proteins normalized to α-tubulin; error bars represent s.e.m.; *n*=4. *P<0.05; **P<0.01; ***P<0.001. α-Tub, α-tubulin; CC, Compound C; Doxy, doxycycline; ns, not significant; Scr, scrambled; sh, shRNA.

Next, we investigated the role of AMPK in the context of physiological stimuli such as hypoxia, a known trigger of EMT (Thiery et al., 2009; Yang et al., 2008). Intriguingly, hypoxia is also a well-known trigger for AMPK activation (Mungai et al., 2011). Based on our data above, we hypothesized that hypoxia-induced EMT might be mediated by AMPK activation. To test this, we exposed epithelial BT474 cells stably expressing either scrambled or AMPKα2 shRNA to the hypoxia mimetic CoCl_2_. Increase in the levels of Hif1α on treatment with CoCl_2_ revealed the generation of a hypoxic environment (Fig. 3A). Consistent with a previous study (Mungai et al., 2011), treatment with CoCl_2_ also triggered an increase in AMPK activity, as revealed by elevated pACC levels (Fig. 3A). Compared with untreated control cells, a decrease in the expression of E-cad and ZO-1 on CoCl_2_ treatment revealed the induction of EMT in scrambled-shRNA-expressing BT474 cells. Interestingly, hypoxia-induced EMT was inhibited upon AMPK knockdown in these cells (Fig. 3A). Immunoblot analysis revealed similar results for partial epithelial-mesenchymal-transitioned A549 cells stably expressing AMPKα2 shRNA and incubated under hypoxic conditions (3% oxygen) in a tri-gas incubator (Fig. 3B; clone #4). Similarly, in mesenchymal MDA-MB-435S cells, an increase in the expression of Vim and N-cad in 3% oxygen revealed the induction of EMT under hypoxia, which was inhibited in the presence of the AMPK inhibitor Compound C (Fig. 3C). Similar results were obtained upon treatment with CoCl_2_ in MDA-MB-231 cells stably expressing AMPKα2 shRNA (Fig. S3A) or in the presence of Compound C (Fig. S3B). These data reveal that AMPK might be required for hypoxia-induced EMT.

Besides hypoxia, another potent inducer of EMT is TGFβ (Polyak and Weinberg, 2009; Yang and Weinberg, 2008). It has previously been shown that treatment of murine hepatocytes and AML12 cells with TGFβ leads to activation of AMPK signaling (Wang et al., 2010). Thus, we speculated that AMPK activity might also be required for TGFβ-induced EMT of cancer cells. To explore this, MDA-MB-435S cells were treated with TGFβ for 48 h. An increase in the protein levels of pSmad2 (Fig. 3D) confirmed the activation of TGFβ signaling in these cells. In keeping with the data of Wang et al., we also saw an increase in AMPK activity upon TGFβ activation, as revealed by an increase in the levels of pACC (Fig. 3D). Furthermore, increased levels of Vim and N-cad revealed the induction of EMT (Fig. 3D). Intriguingly, TGFβ-induced EMT was significantly inhibited in the presence of the AMPK inhibitor Compound C (Fig. 3D). Similar results were obtained upon TGFβ treatment in the presence of Compound C in A549 (Fig. S3C) and MDA-MB-231 cells (Fig. S3D). To further corroborate the data obtained with pharmacological inhibition of AMPK, we addressed the role of AMPK in TGFβ-induced EMT by subjecting MDA-MB-231 cells stably expressing either scrambled or AMPKα2 shRNA to TGFβ treatment for 48 h. An increase in the levels of Vim and N-cad revealed the induction of EMT marker expression. Depletion of AMPK prevented TGFβ-mediated induction of EMT markers at protein levels (Fig. S3E). Together, these data reveal that AMPK might be required for TGFβ-mediated induction of EMT.

Ras signaling has also been shown to promote EMT (Mukherjee, 2010) and increase AMPK activity (Rios et al., 2013). To test whether AMPK is also important for Ras-induced EMT, we inhibited AMPK in HMLER breast cancer cells (Elenbaas et al., 2001) using Compound C. A reduction in the levels of pAMPK and pACC revealed the effects of Compound C (Fig. S3F). We observed that inhibition of AMPK led to a decrease in the expression of mesenchymal markers such as Vim and N-cad while increasing the expression of the epithelial marker E-cad in HMLER cells (Fig. S3F). Thus, collectively these results indicated a critical role for AMPK in the induction of EMT by various upstream stimuli.

**Fig. 4.**
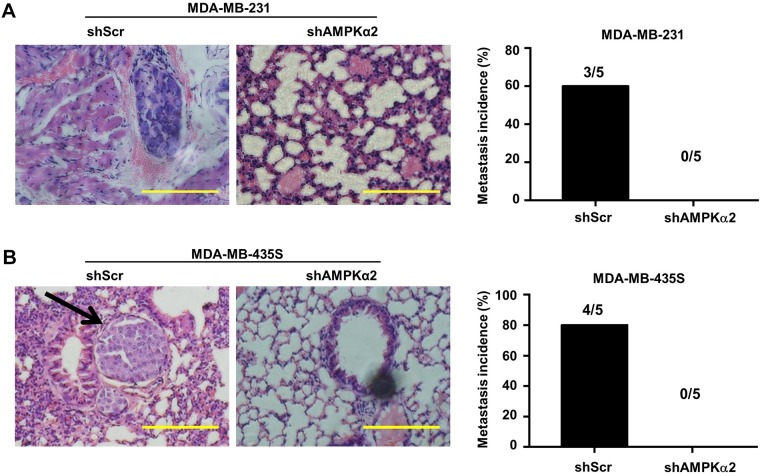
**AMPK knockdown impairs metastasis of breast cancer cells.** (A,B) MDA-MB-231 and MDA-MB-435S cells stably expressing scrambled shRNA or AMPKα2 shRNA were injected through the tail vein of nude mice and scored for lung metastasis. Photomicrographs show histologic sectioning of representative lung metastases originating from MDA-MB-231 (A) and MDA-MB-435S (B) cells (magnification ×20; hematoxylin and eosin staining). Scale bars: 30 μm. Graphs represent incidence of lung metastases found in mice in experiments A and B; *n*=5 mice for each group. Scr, scrambled; sh, shRNA.

Because our study highlighted the role of AMPK in EMT induction, and EMT is considered to be a prerequisite for metastasis (Polyak and Weinberg, 2009), we next investigated the requirement of AMPK function for metastasis *in vivo*. To do so, we subjected MDA-MB-231 cells stably expressing either scrambled- or AMPKα2-shRNA to the experimental tail vein metastasis assay. While several microscopic metastatic colonies were detected in the lungs of mice injected with scrambled shRNA cells, no metastatic growth was detected in mice injected with AMPKα2 knockdown cells (Fig. 4A, Fig. S4A). Similar results were obtained with MDA-MB-435S cells stably expressing scrambled or AMPKα2 shRNA (Fig. 4B, Fig. S4B). An MTT assay revealed no significant change in cell proliferation when AMPK was depleted (Fig. S4C), together suggesting that AMPK functions might be required for the metastasis of these cancer cells.

**Fig. 5.**
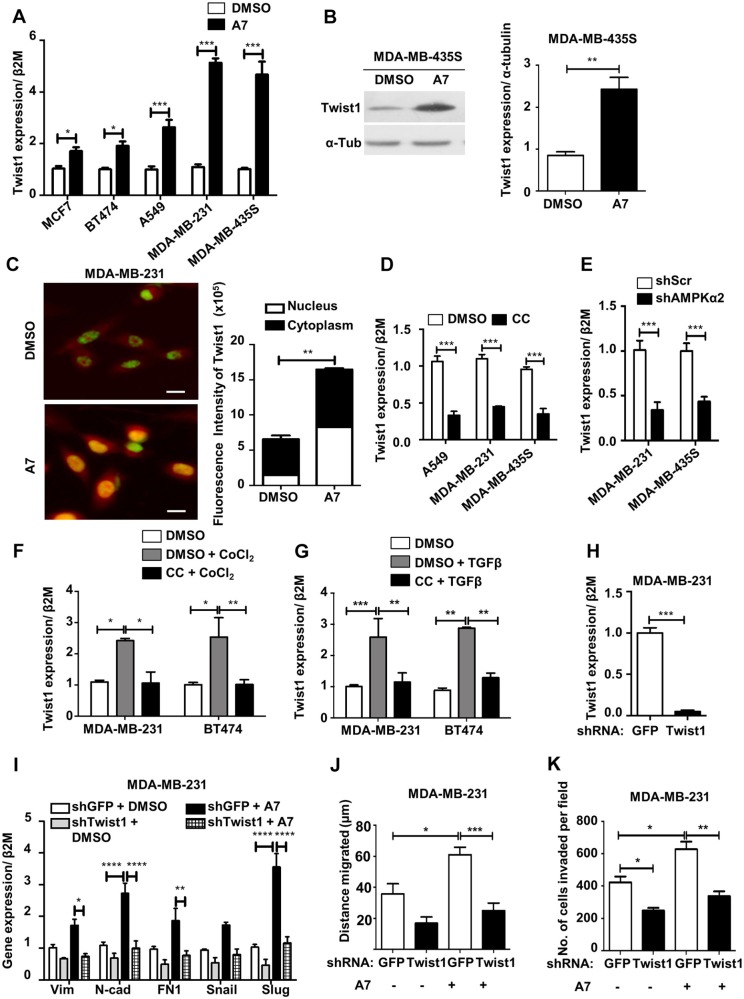
**AMPK regulates Twist1 expression.** (A) Various cancer cell lines (MCF7, BT474, A549, MDA-MB-231 and MDA-MB-435S) were cultured in the presence of 100 µM AMPK activator (A769662) or DMSO (vehicle control) for 48 h. Thereafter, cells were harvested for RNA isolation and subjected to qPCR analysis for Twist1 gene expression. Graph represents fold changes (normalized to housekeeping gene β2M) of Twist1 gene expression in the specified cell lines; error bars represent s.e.m., *n*=3. (B) MDA-MB-435S cells were cultured in the presence of 100 μM AMPK activator (A769662) or DMSO (vehicle control) for 48 h. Thereafter, cells were harvested and subjected to immunoblot analysis for Twist1 and α-tubulin. The graph represents densitometric quantification of the Twist1 protein normalized to α-tubulin; error bars represent s.e.m., *n*=3. (C) MDA-MB-231 cells were cultured in the presence of 100 µM AMPK activator (A769662) or DMSO (vehicle control) for 24 h. Thereafter, the dishes were subjected to immunocytochemistry analysis for Twist1 protein. Photomicrographs show representative fluorescent images taken at 20× magnification using an Olympus 1X71 microscope; Hoechst 33342 was used for nuclear staining, and was pseudo-colored green for representation. Twist1 is stained in red. Scale bars: 10 μm. The graph represents cytoplasmic and nuclear fluorescence intensity (FI) measurements per cell, *n*=30 cells quantified in each of the three biological replicates; the error bar represents s.e.m. (D) qPCR analysis for Twist1 gene expression in A549, MDA-MB-231 and MDA-MB-435S cells cultured in the presence of 10 μM AMPK inhibitor (Compound C) or DMSO (vehicle control) for 48 h. The graph represents fold change (normalized to β2 M); error bars represent s.e.m., *n*=3. (E) qPCR analysis for Twist1 gene expression in MDA-MB-231 and MDA-MB-435S cells stably expressing scrambled shRNA or AMPKα2 shRNA. The graph represents fold change (normalized to β2M); error bars represent s.e.m., *n*=3. (F,G) MDA-MB-231 and BT474 cells were cultured in the presence of 10 µM AMPK inhibitor (Compound C) with 150 µM CoCl_2_ (F) or TGFβ (G) for 48 h. Thereafter, cells were harvested and quantified for Twist1 mRNA levels by qPCR. The graphs represent fold change (normalized to β2M); error bars represent s.e.m., *n*=3. (H) Quantification of Twist1 mRNA levels by qPCR in MDA-MB-231 cells stably expressing GFP shRNA or Twist1 shRNA. The graph represents fold change of Twist1 gene expression (normalized to β2M); error bars represent s.e.m., *n*=3. (I) MDA-MB-231 cells stably expressing GFP shRNA or Twist1 shRNA were cultured in the presence of 100 µM AMPK activator (A769662) or DMSO (vehicle control) for 48 h. Thereafter, the cells were harvested and qPCR analysis was undertaken for the specified transcripts. The graph represents relative gene expression of the specified genes (normalized to β2M expression); error bars represent s.e.m., *n*=4. (J,K) MDA-MB-231 cells stably expressing GFP shRNA or Twist1 shRNA were cultured in the presence of 100 µM AMPK activator (A769662) or DMSO (vehicle control). (J) The graph represents the distance migrated between the cell edges in a scratch assay in 48 h, calculated by Image J software; error bars represent s.e.m., *n*=3. (K) The specified cells treated similarly were also subjected to Boyden chamber invasion assay. The graph represents the total number of cells invaded per field; error bars represent s.e.m., *n*=4. *P<0.05; **P<0.01; ***P<0.001. α-Tub, α-tubulin; A7, A769662; CC, Compound C; Scr, scrambled; sh, shRNA.

Next, we investigated the mechanisms by which AMPK might regulate EMT. Induction of EMT is associated with the upregulation of several transcription factors such as Twist1, Snail, Slug and Zeb1 (Yang and Weinberg, 2008). Of these, Twist1 has been considered to be a major regulator of EMT, invasion and metastasis (Wang et al., 2016; Yang et al., 2004). Therefore, we analyzed the expression of Twist1 under conditions of AMPK activation. We noticed an AMPK-mediated increase in Twist1 transcripts in a variety of cell lines tested, including MCF7, BT474, A549, MDA-MB-231 and MDA-MB-435S cells (Fig. 5A). Notably, the epithelial-mesenchymal-transitioned cells showed a greater increase in Twist1 transcript levels upon AMPK activation compared with the non-invasive cells (Fig. 5A). A western blot analysis revealed elevated Twist1 protein levels in the presence of A769662 (Fig. 5B and Fig. S5A). An immunocytochemical analysis further confirmed elevated Twist1 levels (Fig. 5C).

To validate whether Twist1 regulation is through AMPK activation, we next investigated the effects of AMPK inhibition on Twist1 expression in the epithelial-mesenchymal-transitioned cancer cells lines A549, MDA-MB-231 and MDA-MB-435S. Indeed, in these cell lines, inhibition of AMPK with Compound C led to a significant decrease in the levels of Twist1 transcripts (Fig. 5D). Consistent with this, MDA-MB-231 and MDA-MB-435S cells stably expressing AMPKα2 shRNA also showed less expression of Twist1 compared with scrambled cells (Fig. 5E). Importantly, inhibition of AMPK prevented the induction of Twist1 expression by CoCl_2_ or TGFβ treatment (Fig. 5F,G). Taken together, these data suggested that AMPK might mediate its effects on EMT through upregulation of Twist1.

To gauge the role of Twist1 downstream of AMPK in the process of EMT, we investigated the effects of Twist1 depletion in AMPK-mediated EMT response of cancer cells. For this, we generated MDA-MB-231 cells stably expressing Twist1 shRNA (Mani et al., 2008). These cells showed ∼90% reduction of Twist1 both at transcript level (Fig. 5H) and protein level (Fig. S5B,C) compared with control shGFP cells. Consistent with the literature (Mani et al., 2008), Twist1 knockdown led to a reduction in the expression of EMT markers such as Vim, N-cad, fibronectin 1 (FN1; also known as CIG), Snail and Slug (Fig. 5I). As shown previously, AMPK activation with A769662 led to increased EMT marker expression in the control shGFP cells; however, AMPK activation failed to do so in the Twist1 knockdown cells (Fig. 5I). Similar results were obtained in MDA-MB-435S cells expressing Twist1 siRNA oligos (Fig. S5D,E), indicating that AMPK might mediate its effects on EMT, at least in part, through Twist1.

Furthermore, to determine whether AMPK requires Twist1 for mediating functional changes associated with EMT, we investigated the effects of AMPK activation in the migration and invasion of Twist1 knockdown cells. As seen before, AMPK activation led to an increase in the migration and invasive potential of control shGFP cells (Fig. 5J,K and Fig. S5F); however, AMPK activation in shTwist1 cells failed to do so (Fig. 5J,K and Fig. S5F).

**Fig. 6.**
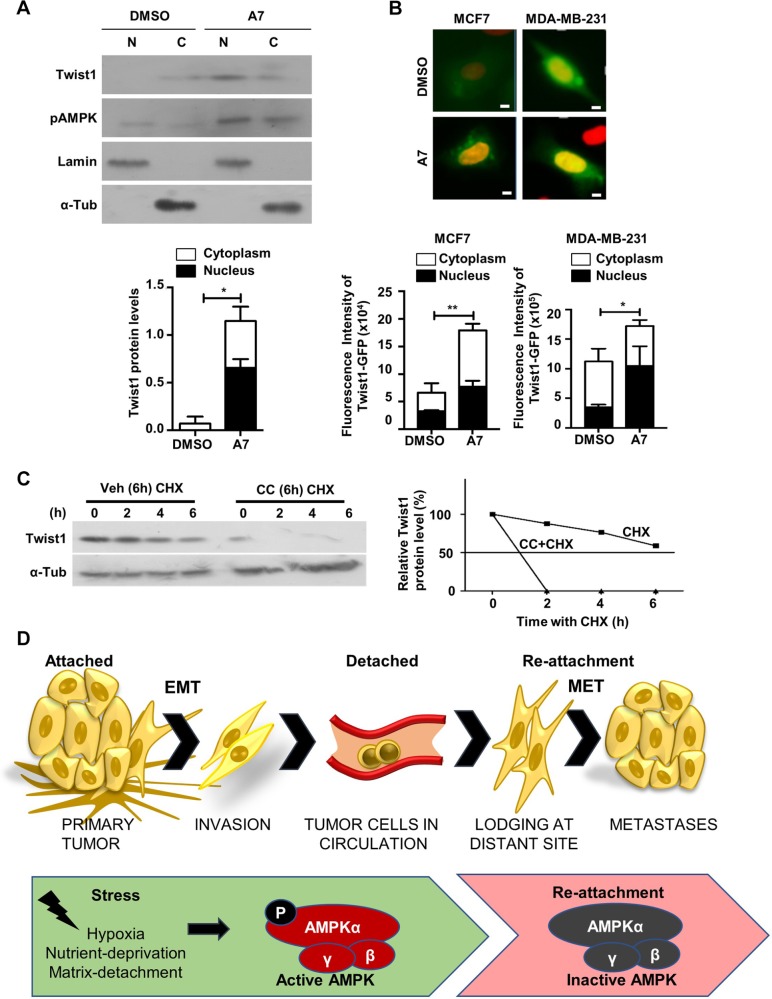
**AMPK mediates EMT via increased Twist1 stability and its nuclear translocation.** (A) MDA-MB-231 cells were cultured in the presence of 100 μM AMPK activator (A769662) or DMSO (vehicle control) for 48 h. Thereafter, cells were harvested, cytoplasmic and nuclear proteins were fractionated and subjected to immunoblot analysis for Twist1, pAMPKα, lamin and α-tubulin, *n*=3. The graph represents densitometric quantification of nuclear and cytoplasmic Twist1 levels; error bars represent s.e.m., *n*=4. *P<0.05. (B) MCF7 and MDA-MB-231 cells were transiently transfected with Twist1 tagged to GFP and subsequently cultured in the presence of 100 µM AMPK activator (A769662) or DMSO (vehicle control) for 24 h. Thereafter, the cells were fixed and localization of GFP fluorescence was analyzed. Photomicrographs show representative fluorescent images taken at 20× magnification using an Olympus 1X71 microscope; Hoechst 33342 was used for nuclear staining, and was pseudo-colored red for representation. Twist1-GFP is in green. Scale bars: 10 μm. The graphs represent nuclear and cytoplasmic localization of Twist1-GFP, *n*=30 cells quantified in each of the three biological replicates; error bars represent s.e.m. *P<0.05; **P<0.01. (C) MDA-MB-435S cells were cultured in the presence of 10 μM AMPK inhibitor (Compound C) or DMSO (vehicle control) for 6 h followed by inhibition of protein translation by cycloheximide treatment for 0, 2, 4 and 6 h. Cells were harvested and subjected to immunoblot analysis for Twist1 and α-tubulin. The graph represents densitometric quantification of Twist1 levels normalized to α-tubulin. (D) Model: Stresses faced by tumor cells in the primary tumor environment, such as hypoxia, nutrient deprivation and matrix detachment, activate AMPK, which in turn induces EMT, allowing tumor cells to initiate the invasion-metastasis cascade. Re-attachment at distant sites causes inhibition of AMPK signaling, enabling reversal of EMT and establishment of secondary tumors. α-Tub, α-tubulin; A7, A769662; CC, Compound C; CHX, cycloheximide.

Because Twist1 is a transcription factor and its nuclear localization is critical for its function, we investigated nuclear localization of Twist1 under AMPK-activated conditions. As seen in the immunofluorescence images (Fig. 5C), in addition to increased levels of Twist1, we also noted increased nuclear localization of Twist1 in the AMPK-activated condition. Because AMPK activation led to a significant increase in Twist1 in MDA-MB-231 cells (Fig. 5A and Fig. S5A), we performed subcellular fractionation experiments with the same cells. Cell fractionation experiments also showed elevated nuclear Twist1 upon AMPK activation (Fig. 6A). Furthermore, immunofluorescence imaging of MDA-MB-231 and MCF7 cells transiently transfected with a GFP-tagged Twist1 construct also showed increased nuclear localization of Twist1 in the A769662-treated condition (Fig. 6B), suggesting AMPK-mediated post-translational modifications of Twist1 leading to its nuclear localization. Because phosphorylation by mitogen-activated protein kinases (MAPKs), extracellular signal-regulated kinases (ERKs) and Jun N-terminal kinases (JNKs) has been shown to increase serine phosphorylation and stability of Twist1 (Hong et al., 2011b), we investigated the effect of AMPK activation on Twist1 stability. In cycloheximide chase experiments, inhibition of AMPK led to a decrease in Twist1 stability (Fig. 6C). Moreover, we observed increased Twist1 stability in the presence of AMPK activator A769662 (data not shown). Taken together, our data revealed that AMPK activation leads to an increase in the expression, stability and nuclear localization of Twist1 protein, which ultimately could promote EMT.

In conclusion, our study uncovers a central role for AMPK in the positive regulation of EMT in cancer cells, mediated, at least in part, through Twist1 upregulation.

## DISCUSSION

EMT is suggested to be an initial and important step in the invasion-metastasis cascade (Thiery et al., 2009). Therefore, an increased understanding of the molecular players involved in this process is likely to lead to a better understanding of cancer metastasis and identify newer targets for cancer treatment. Although several signaling pathways and physiological conditions have been identified as activators of EMT, and a plethora of transcription factors have been identified as downstream effectors of EMT, it has remained unclear whether these various inducers of EMT function independently, work in conjunction, or converge on a common downstream mediator. Our study identifies a central role for AMPK in mediating an EMT downstream of several physiological cues. Even though the LKB1-AMPK axis was initially associated with tumor-suppressive roles (Huang et al., 2008), recent studies have demonstrated pro-tumorigenic functions for AMPK, including inhibition of apoptosis, promoting anchorage-independent growth and, more recently, cancer cell migration (Hindupur et al., 2014; Jeon and Hay, 2015; Kim et al., 2011; Ng et al., 2012; Wang et al., 2010). In our study, we provide evidence for a direct role of AMPK in the induction of EMT in cancer cells, as well as in the maintenance of the EMT phenotype. We further show that activation of AMPK suffices to bring about a functional EMT characterized by increased migration and invasion potential. Moreover, we show that AMPK activation is mandatory for EMT induction mediated by upstream physiological cues such as hypoxia and TGFβ. Additionally, we show that AMPK mediates its effects on EMT via upregulation of *TWIST1* gene expression and its increased nuclear localization.

AMPK activation with A769662 affected the expression of epithelial markers E-cad and ZO-1 to different extents in different cell lines promoting an EMT. In the epithelial breast cancer cells MCF7, T47D and BT474, AMPK activation decreased the expression of epithelial markers E-cad and ZO-1 and caused dissolution of adherens junctions from the cell membrane (the first hallmark of an active EMT), thus pushing the cells towards initiating an EMT. Furthermore, even though AMPK activation upregulated the transcript levels of mesenchymal markers (Vim and N-cad) in these cells, their protein levels are not changed (data not shown), and consistent with this, their migratory properties also did not change. Hence, these cells seem to only ‘partially’ undergo an EMT. In metastable A549 cells, which already seem to be in a partial EMT state as evident by E-cad^low^ and Vim^low^ phenotype, AMPK activation significantly decreased the protein expression of E-cad and ZO-1 and upregulated mesenchymal markers (Fig. S6). Thus, metastable A549 cells, which seem to be ‘primed’ for EMT, are hence pushed further into EMT in response to AMPK activation. Mesenchymal MDA-MB-231 cells do not express E-cad while expressing ZO-1 at a low basal level. AMPK activation was able to further downregulate ZO-1 expression in these mesenchymal cells, and significantly increased the expression of mesenchymal markers (Fig. S6) and their migratory and invasive potential. Hence, it appears that although AMPK activation is able to downregulate the expression of epithelial markers across all states of EMT in different cell types, additional cues might be required to also increase the protein expression of mesenchymal markers in epithelial cells (MCF7, T47D and BT474) to bring about a complete, functional EMT. In contrast, primed partial epithelial-mesenchymal-transitioned cells (A549) and mesenchymal cells (MDA-MB-231 and MDA-MB-435S) seem to already possess the requisite machinery to bring about a complete, functional EMT in response to AMPK activation.

On the other hand, AMPK inhibition or depletion increased the epithelial characteristics of the cells while decreasing mesenchymal properties. In the epithelial BT474 cells, AMPK knockdown led to a further increase in epithelial markers (Fig. S6). In the partially epithelial-mesenchymal-transitioned A549 cells, AMPK inhibition or knockdown led to an increase in epithelial markers E-cad and ZO-1 in addition to reduction in mesenchymal markers Vim and N-cad. AMPK inhibition or depletion in the mesenchymal MDA-MB-231 and MDA-MB-435S cell lines decreased the expression of mesenchymal markers Vim and N-cad (Fig. S6); however, it failed to bring back E-cad expression (data not shown). It is known that the E-cad promoter is hypermethylated in MDA-MB-435S cells, completely silencing E-cad expression, whereas it is partially methylated in MDA-MB-231 cells, leading to a very low expression of E-cad that cannot be detected by western blotting (Lombaerts et al., 2006). It is possible that AMPK signaling may not be able to alleviate such hypermethylation-induced gene silencing. These data suggested that AMPK is required to maintain the EMT phenotype, whereas its inhibition leads to reversal of EMT, also known as mesenchymal-to-epithelial transition (MET) (Nieto et al., 2016).

Our data revealed that activation of AMPK using A769662 promotes EMT. In contrast, metformin, also an AMPK activator, has been shown to inhibit the EMT phenotype of breast cancer cells (Qu et al., 2014). Another study showed that activation of AMPK by OSU-53, a new allosteric activator of AMPK, reversed EMT (Chou et al., 2014). Metformin was shown to inhibit EMT by AMPK-mediated abrogation of Erk activity, leading to downregulation of Snail and Slug in lung and breast cancer cells (Banerjee et al., 2016). AMPK signaling has been shown to inhibit EMT in other cancer types such as melanomas, lung and prostate cancer (Chou et al., 2014; Kim et al., 2012; Lin et al., 2015). These discrepancies could be due to the extent of AMPK activation by different pharmacological agents, or due to their non-specific activities. Metformin, a biguanide used as a first-line anti-diabetic drug, is an indirect modulator of AMPK. It inhibits complex I of the mitochondrial respiratory chain, causing an increase in intracellular AMP levels, thereby activating AMPK (Owen et al., 2000). However, AMPK-independent roles of metformin have been reported (Foretz et al., 2010; Kalender et al., 2010) and its use as a bona fide AMPK activator needs further investigation. A769662 and AICAR, both analogs of AMP, bind directly to the auto-inhibitory domain of AMPK and do not cause a change in the ATP:ADP ratio or alter mitochondrial function to exert their action. However, AICAR monophosphate (ZMP), the active form of AICAR within cells, is a natural intermediate of the purine nucleotide synthesis pathway and is metabolized by AICAR transformylase (Kim et al., 2016). Therefore, the turnover of the active form of AICAR within cells is dependent on their proliferation rate. Thus, these modulators of AMPK might activate it to different extents. Our results with A769662 showed that AMPK is a positive regulator of EMT in multiple cancer cell lines. We have additionally corroborated our results with genetic approaches using either siRNA- or inducible-shRNA-mediated knockdown.

Most importantly, our data revealed that TGFβ, hypoxia, and oncogenic Ras-mediated EMT are significantly inhibited by depletion or inhibition of AMPK. This suggested that activation of AMPK could be a key central event in the EMT program. Recent studies have identified critical functions of AMPK under pathological/physiological stresses associated with tumor progression, including nutrient deprivation, hypoxia, and matrix detachment (Hindupur et al., 2014; Steinberg and Kemp, 2009). Our identification of a stress-response kinase as a necessary component of the EMT program additionally supports the notion that the phenomenon of EMT might be a stress response that aids cancer cell dissemination and metastasis. Furthermore, given that the earlier studies have linked the attainment of an EMT phenotype with the acquisition of stemness properties (Mani et al., 2008), it is plausible that AMPK activity may determine the differentiation status of epithelial cancers.

EMT is largely effected by a set of transcription factors whose expression and function are under tight control. Twist1, considered as a master regulator of EMT and metastasis (Yang et al., 2004), is expressed in a variety of cancers (Ansieau et al., 2010). Several transcriptional regulators of Twist1 have been identified, including HIF1α, interleukin-6 (IL-6), NF-κB and the Smad-dependent TGFβ pathway (Cheng et al., 2008; Pham et al., 2007; Tan et al., 2012). Our study showed that Twist1 gene expression is transcriptionally regulated by AMPK. Indeed, AMPK has been shown to regulate gene expression by phosphorylating various transcription factors (Cantó and Auwerx, 2010), or via its effects on chromatin structure by modulating histone deacetylase activity or histones (Bungard et al., 2010; McGee et al., 2008). However, the precise mechanism of Twist1 transcriptional regulation by AMPK remains to be elucidated.

Additionally, our study revealed that AMPK activation led to increased stability and nuclear localization of Twist1. Such a multi-step regulation of Twist1 by AMPK suggests that the cellular events downstream of the AMPK-Twist1 axis play a critical role in orchestrating the EMT program. Twist1 is also known to be phosphorylated by kinases such as Akt and MAPK, which affect its anti-apoptotic function and stability (Hong et al., 2011b; Vichalkovski et al., 2010). Although Twist1 possesses an AMPK consensus motif (data not shown), AMPK-mediated phosphorylation of Twist1 is yet to be specified. Interestingly, we also observed a significant increase or decrease in levels of other EMT transcription factors such as Snail, Slug and Zeb1 on AMPK activation or inhibition, respectively. Furthermore, like Twist1, Snail and Slug also possess an AMPK consensus motif in their protein sequence (data not shown), suggesting that AMPK might additionally regulate these EMT transcription factors by phosphorylating them. Hence, although in the present study we have investigated the AMPK-Twist1 axis, we speculate that AMPK might also be able to regulate EMT mediated by other transcription factors.

The eventual outgrowth of metastatic colonies at a secondary organ site is the culmination of a series of steps referred to as the invasion-metastasis cascade. EMT is proposed to be one of the early steps that helps initiate this cascade, enabling the epithelial cells at the primary tumor site to undergo morphogenetic changes and assume a mesenchymal morphology that enables the cancer cells to migrate and invade following detachment from the extracellular matrix. In fact, previous studies have shown that AMPK is activated on detachment from the matrix (Hindupur et al., 2014; Jeon and Hay, 2012), supporting our finding that AMPK activation leads to EMT. After traversing through blood/lymphatic vessels, the epithelial-mesenchymal-transitioned cancer cells extravasate at a secondary site, revert their morphology to an epithelial phenotype, re-attach to the matrix, and then undergo a clonal outgrowth called micro-/macro-metastasis. This process is referred to as MET (Nieto et al., 2016). Indeed, we have previously shown that re-attachment to the matrix downmodulates AMPK activity (Sundararaman et al., 2016), suggesting that at later stages of metastasis, AMPK might be inactivated, causing MET and thus helping the formation of a new tumor growth at a secondary site. This is consistent with our observation that AMPK inhibition leads to a reduction in EMT markers. Thus, matrix-adhesion state could regulate AMPK activity, which in turn could regulate EMT and MET reversibly, thus enabling metastasis (Fig. 6D).

Taken together, our study elicits an important role for AMPK in tumor progression by regulating EMT, migration and invasion of breast cancer cells. The requirement of AMPK downstream of several cues that are known activators of EMT in cancer cells suggests a possible central role for AMPK in orchestrating the EMT program. Targeting AMPK might thus serve as a novel therapeutic strategy to control cancer metastasis. Because AMPK plays a major role in maintaining energy homeostasis under metabolically stressed conditions, its inhibition for a therapeutic window with minimal systemic effects might be exploited for cancer treatment in the future with the development of specific AMPK inhibitors for clinical use.

## MATERIALS AND METHODS

### Cell culture and reagents

MDA-MB-231, MCF7, T47D (breast epithelial cancer cell lines), MDA-MB-435S (melanoma) and A549 (lung cancer) cells were cultured in DMEM (Sigma-Aldrich, St. Louis, MO) supplemented with 10% FBS, penicillin (1 kU/ml) and streptomycin (0.1 mg/ml). BT474 (breast cancer) cells were cultured in RPMI (Sigma-Aldrich) supplemented with 10% FBS, penicillin (1 kU/ml) and streptomycin (0.1 mg/ml). HMLER cells (provided by Prof. Robert Weinberg, Department of Biology, Massachusetts Institute of Technology, Cambridge, MA) were cultured in DME-F12 medium supplemented with hEGF (10 ng/ml), hydrocortisone (0.5 μg/ml) and insulin (10 μg/ml).

Pharmacological chemicals used in this study include A769662 (100 µM; purchased from University of Dundee, Dundee, UK), 6-[4-(2-piperidin-1-ylethoxy-phenyl)]-3-pyridin-4-yl-pyrrazolo [1,5-a]-pyrimidine (Compound C) (10 µM; Calbiochem, Gibbstown, NJ) and cycloheximide (100 μg/ml; Sigma-Aldrich). Absolute dimethyl sulfoxide (DMSO) (Calbiochem) was used as a vehicle for A769662 and Compound C. Recombinant human TGFβ (R&D Systems, Minneapolis, MN) was used at a concentration of 5 ng/ml. For hypoxia-based experiments, cells were either cultured in 3% oxygen using a tri-gas incubator (Thermo Fisher, Waltham, MA) or treated with 150 μM cobalt chloride.

### Antibodies and immunoblotting

Whole cell lysates for western blotting were prepared with lysis buffer containing 1% NP-40 detergent, 0.5% sodium deoxycholate, 0.1% SDS, 50 mM sodium fluoride, 1 mM sodium orthovanadate, 10 mM sodium pyrophosphate (Sigma-Aldrich) and protease inhibitors (Roche, Mannheim, Germany). Protein concentration was estimated with Bradford reagent and an equal amount of protein (25 μg) was resolved by SDS-PAGE using Bio-Rad apparatus, transferred to a PVDF membrane (Millipore, Billerica, MA) and probed with appropriate antibodies. For all western blots involving phospho-protein analysis, the levels of respective total proteins were probed and shown to be equal. α-Tubulin (Calbiochem) served as a loading control. HRP-coupled secondary antibodies were obtained from The Jackson Laboratory, and immunoblots were visualized using PICO reagent (Pierce, Waltham, MA). Primary antibodies used were: phospho-AMPK (Thr172), total AMPKα2, phospho-ACC, total ACC (recognizes both isoforms 1 and 2), phospho-Smad2, total Smad2 and vimentin (Cell Signaling Technology, Beverly, MA); N-cadherin and E-cadherin (Epitomics, Burlingame, CA); Twist1 (Sigma-Aldrich); and HIF1α (Upstate, Billerica, MA). Multiple panels for a given experiment were developed from the same blot by re-probing. Alternatively, the same lysates were loaded as technical replicates; for each technical run, α-tubulin was probed as a loading control. All primary antibodies were used at 1:1000 dilution in 5% BSA, whereas HRP-conjugated secondary antibodies were used at 1:3000 dilution.

Quantitative densitometric analysis of the EMT-related immunoblots was undertaken using ImageJ software. Protein expression was normalized to α-tubulin; the normalized expression of control was set to 1, and an increase or decrease in expression under various conditions was calculated relative to the control.

### Nuclear-cytoplasmic fractionation

For fractionation experiments MDA-MB-435S cells were grown in 90-mm dishes until they reached 80% confluence. The cells were then treated with A769662 for 2 h and lysed in mild lysis buffer [0.5% NP-40, 10 mM sodium pyrophosphate, 1 mM sodium orthovanadate, and 1× protease inhibitor (Roche) diluted in PBS]. The supernatant obtained after spinning the lysate served as the cytoplasmic fraction. The pellet obtained was gently washed twice with 1× PBS, subjected to a freeze-thaw cycle at −80°C and then resuspended in western lysis buffer cocktail. The supernatant obtained after spinning the lysate served as the nuclear fraction. The cytoplasmic and the nuclear fractions were electrophoresed on an SDS-PAGE gel and subsequently subjected to immunoblotting.

### Transfection

All plasmid transfections were performed using Lipofectamine 2000 (Invitrogen, Carlsbad, CA) as per the manufacturer's instructions. Twist shRNA was provided by Prof. Sendurai Mani (MD Anderson Cancer Center, Houston, TX).

### RNAi experiments

AMPKα2 knockdown stable cells were generated by transfecting BT474, MDA-MB-435S and MDA-MB-231 (Hindupur et al., 2014) cells with a pool of four shRNA constructs (HuSH-29 shRNA vectors; Origene, Rockville, MD) targeting AMPKα2. Stable cells were generated using puromycin selection followed by sorting for RFP expression (encoded by the vector) and were expanded and frozen for future use. Knockdown was confirmed by immunoblotting. Simultaneously, all of the cell types were transfected with scrambled shRNA and stable cells were generated as above. MDA-MB-231 and BT474 cells with a stable knockdown of AMPKα2 were validated and described previously (Hindupur et al., 2014).

Inducible AMPKα2 knockdown cells were generated by transfecting the pTRIPZ vector expressing shRNA against AMPKα2 under the Tet-On promoter [Seq no. 1: V2THS_57638 (Clone #1); Seq no. 4: V2THS_375319 (Clone #4); Dharmacon, Pittsburgh, PA] in A549 and MDA-MB-231 cells. Stable transfectants were selected using puromycin, followed by sorting for RFP (encoded by the vector) after induction with doxycycline (5 µg/ml) for 48 h. Knockdown was confirmed using immunoblotting. For further experiments, uninduced stable cells were used as control.

For siRNA transfections, cells were seeded in 60-mm dishes at 50% confluence and were transfected with non-targeting control siRNA (Sigma-Aldrich), Twist1 siRNA (Sigma-Aldrich) or AMPKα2 siRNA (Cell Signaling Technology) using Oligofectamine (Invitrogen) as per the manufacturer's instructions.

### RNA extraction and RT-PCR

Total RNA was isolated using TRI reagent (Sigma-Aldrich). Reverse transcription of mRNA was carried out using a Gene-Amp RNA PCR cDNA synthesis kit (Applied Biosystems, Carlsbad, CA). Specific primers (Table S1) were designed using Primer 3.0 software. Hypoxanthine-guanine phosphoribosyltransferase (HPRT) or β2 microglobulin (β2M) were used as normalizing control.

### Wound-healing scratch assay

Migration assay was performed using an ImageLock 96-well plate in the IncuCyte ZOOM live-cell analysis system (Essen Bioscience), where 2×10^4^ of control and knockdown of MDA-MB-231, BT474 and A549 cells were seeded into each well and treated with 10 µg/ml of Mitomycin C (Calbiochem) for 2 h. A scratch wound was made using WoundMaker™ (Essen Bioscience). Cells were then washed with 1× PBS and incubated with culture medium without serum and imaged every 1 h. The extent of wound healing was determined as wound confluence percentage using IncuCyte software (Essen Bioscience). Each individual experiment had three to five technical replicates.

Independently, an equal number of MDA-MB-435S cells were seeded in 60-mm dishes. siRNA transfection was performed for 48 h. Cells were treated with 10 μg/ml Mitomycin C (Calbiochem) for 2 h to arrest proliferation, following which, two wounds were made using a P-200 pipette tip. Photomicrographs were taken at 0 and 48 h of wound generation. The distance migrated was quantified using ProgRes capture software and plotted as a difference of distance migrated between 0 and 48 h.

### ECIS wound-healing assay

Real-time quantitative wound-healing assays were performed using the Electric Cell-substrate Impedance Sensing (ECIS; model 1600R; Applied BioPhysics, Troy, NY) and the 8W10E ECIS arrays. MDA-MB-435S cells were treated with A769662 or Compound C for 48 h followed by treatment with Mitomycin C for 2 h to arrest proliferation, trypsinized and a total of 2×10^5^ live cells seeded in three wells of the 8W10E ECIS array. One well with only medium (no cells) served as a negative control to provide the baseline changes in impedance. Cells were allowed to attach for 24 h, after which a wound was made using an elevated AC voltage pulse with a frequency of 40 kHz, and duration of 30 s. Dead cells were washed away by replacing the medium. Migratory response over the next 24 h was measured in real time by recording the recovery of electrical impedance.

### Matrigel invasion assay

MDA-MB-231 and MDA-MB-435S cells treated with A769662 or Compound C for 48 h were trypsinized and a total of 50,000 live cells were seeded in the BD BioCoat™Matrigel™ invasion chambers. DMSO served as the vehicle control. After 24 h, the non-invaded cells were removed, and the invaded cells were fixed with 4% paraformaldehyde and stained with Crystal Violet Blue. Photomicrographs of the invasion chambers were taken at 10× magnification. The number of invaded cells was counted from these images using ImageJ software and plotted as a graph. MDA-MB-231 (2×10^4^ cells) and MDA-MB-435S (5×10^4^) cells stably expressing control shRNA or AMPKα2 shRNA were similarly subjected to Matrigel invasion assays.

### Metastasis assay

A total of 1×10^6^ cells (control or AMPKα2 knockdown cells) in 50 µl of DMEM were injected into the lateral vein of nude mice. The mice were euthanized after 9 weeks of injection. The lungs were dissected out, fixed in 4% paraformaldehyde overnight and embedded in paraffin. Hematoxylin and eosin staining was undertaken on multiple lung sections and scored by a pathologist for the detection of metastatic foci. Animal experiments were performed in compliance with CPCSEA guidelines.

### Immunofluorescence

Various cancer cell lines or stable cells generated were treated with A769662 (100 µM), Compound C (10 µM) or vehicle control DMSO for 24 or 48 h as mentioned earlier and fixed with 4% paraformaldehyde at room temperature for 2 h. The cells were permeabilized with PBS containing Triton-X 100, blocked with 1% BSA and probed with antibodies (1:200 dilution) at 4°C overnight. The cells were probed with secondary antibody tagged with FITC or Cy3 (1:200 dilution) for 2 h at room temperature. The cells were then washed, mounted and visualized under an epifluorescence microscope (Olympus IX71). Further image processing and quantification was performed using ImageJ software. For relative fluorescence intensity (RFI) quantification, fluorescence intensity was first normalized to the number of nuclei; the normalized intensity of control was set to 1, and the change in intensity under various conditions was plotted relative to the control.

### Proliferation assay

MDA-MB-231 (5×10^3^), BT474 (8×10^3^) and A549 (5×10^3^) cells expressing scrambled shRNA or AMPKα2 shRNA were seeded into a 96-well plate to measure their proliferation rates. Cells were seeded in triplicates for each time point (day 0 to day 4). At each time point, 20 µl of MTT reagent (5 mg/ml) was added to each well of the plate and incubated for 4 h. The incubation medium was then removed and 100 µl of DMSO was added to each well to dissolve the formazan crystals, then absorbance was measured using a plate reader at 570 nm. A proliferation rate graph was plotted using GraphPad Prism software.

### Determination of protein stability

For Twist1 protein stability experiments following AMPK inhibition, MCF7 cells were seeded in eight 60-mm dishes at 30-40% confluence, and transfected with 1 μg of pcDNA3-Flag Twist1 each from a common DNA-transfection reagent mix. After 24 h of seeding, four dishes were treated with Compound C for 6 h, following which, 100 μg/ml cycloheximide was added to all of the dishes. Compound-C-treated and untreated dishes were harvested at 0, 2, 4 and 6 h of cycloheximide treatment for immunoblotting.

### Statistics

All statistical analysis was performed using ratio *t*-test one-way ANOVA and two-way ANOVA with GraphPad Prism 5.0 software. All data were presented as means±s.e.m. of three independent experiments unless otherwise stated. *P*<0.05 was considered to be statistically significant.

## Supplementary Material

Supplementary information
