# Supplementary material for: AMP-activated protein kinase promotes epithelial-mesenchymal transition in cancer cells through Twist1 upregulation
Source: J Cell Sci. 2018 Jul 26;131(14):jcs208314. doi: 10.1242/jcs.208314 (PMC6080604; doi:10.1242/jcs.208314)
Supplement: Supplementary information [file joces-131-208314-s1.pdf]

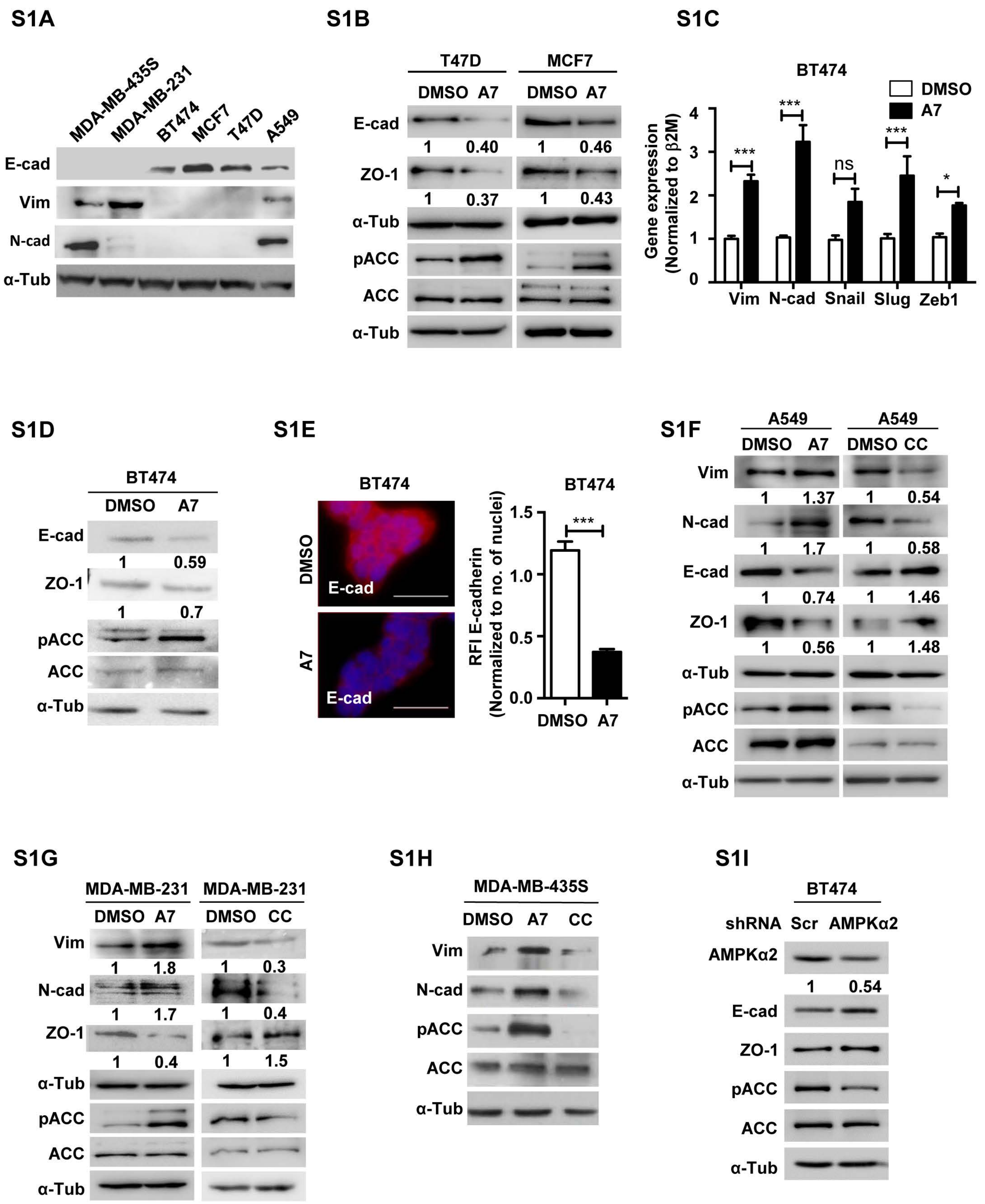

Journal of Cell Science • Supplementary information

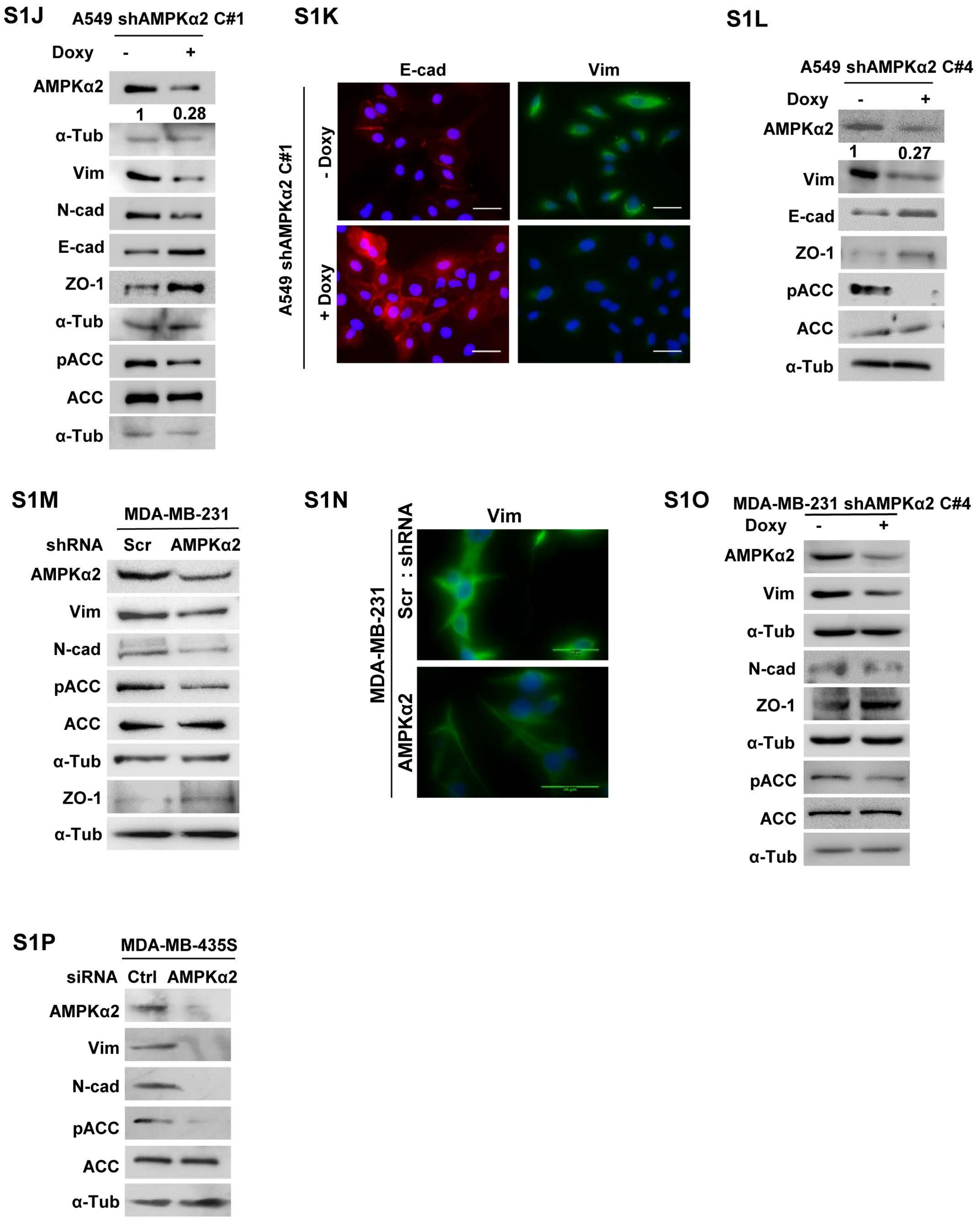

Journal of Cell Science • Supplementary information

Figure S1:

(S1A) Cancer cell lines MDA-MB-435S, MDA-MB-231, BT474, MCF7, T47D and A549 were subjected to immunoblot analysis for the detection of endogenous levels of the specified proteins, n=3.

(S1B) T47D and MCF7 cells were cultured in the presence of 100 µM AMPK activator (A769662) or DMSO (vehicle control) for 48 hours, followed by immunoblot analysis for the specified proteins, n=4. Band intensities are represented below the relevant panels.

(S1 C, D and E) BT474 cells were cultured in the presence of 100 µM AMPK activator (A769662) or DMSO (vehicle control) for 48 hours. Thereafter, qPCR analysis **(C)** was undertaken for the specified transcripts, n=3. Parallel dishes were subjected to immunoblot analysis for the specified proteins, n=3 **(D)**, and immunocytochemical analysis for E-cadherin protein **(E)**. Photomicrographs show representative fluorescent images taken at 20X magnification using Olympus 1X71 microscope; Hoechst 33342 was used for nuclear staining. Graph represents relative fluorescence intensity (RFI) measurements normalized to number of nuclei, n=3.

(S1 F and G) A549 (**F**) and MDA-MB-231 (**G**) cells were cultured in the presence of 100 µM AMPK activator (A769662), 10 µM AMPK inhibitor (Compound C) or DMSO (vehicle control) for 48 hours, followed by immunoblot analysis for the specified proteins; n=4. Same DMSO treated samples were used as a control for A769662 or Compound C treated samples, but developed with different exposures to capture the differences. Band intensities are represented below the relevant panels.

(S1H) MDA-MB-435S cells were cultured in the presence of 100 µM AMPK activator (A769662), 10 µM AMPK inhibitor (Compound C) or DMSO (vehicle control) for 48 hours followed by immunoblot analysis for the specified proteins; n=4.

(S1I) BT474 cells stably expressing scrambled shRNA or AMPKα2 shRNA were harvested and subjected to immunoblot analysis for the specified proteins, n=2.

(S1J and K) A549 cells stably expressing inducible shRNA against AMPKα2 (clone#1) cultured with and without doxycycline for 48 hours, were harvested and subjected to immunoblot analysis for the specified proteins, n=2 **(J)**. Parallel dishes were subjeted to immunocytochemical analsysis for E-cadherin and vimentin protein, n=2 **(K)**. Photomicrographs show representative fluorescent images taken at 20X magnification using Olympus 1X71 microscope; Hoechst 33342 was used for nuclear staining.

(S1L) A549 cells stably expressing inducible shRNA against AMPKα2 (clone#4) cultured with and without doxycycline for 48 hours, were harvested and subjected to immunoblot analysis for the specified proteins, n=2

(S1M and N) MDA-MB-231 cells stably expressing scrambled shRNA or AMPKα2 shRNA were harvested and subjected to immunoblot analysis for the specified proteins, n=3 (**K**). Parallel dishes were subjected to immunocytochemistry analysis for Vimentin, n=3 (**L**).

(S1O) MDA-MB-231 cells stably expressing inducible shRNA against AMPKα2 (clone #4) cultured with and without doxycycline for 48 hours, were harvested and subjected to immunoblot analysis for the specified proteins, n=2.

(S1P) MDA-MB-435S cells transfected with control siRNA or AMPKα2 siRNA for 48 hours were harvested and subjected to immunoblot analysis for the specified proteins; n=4.

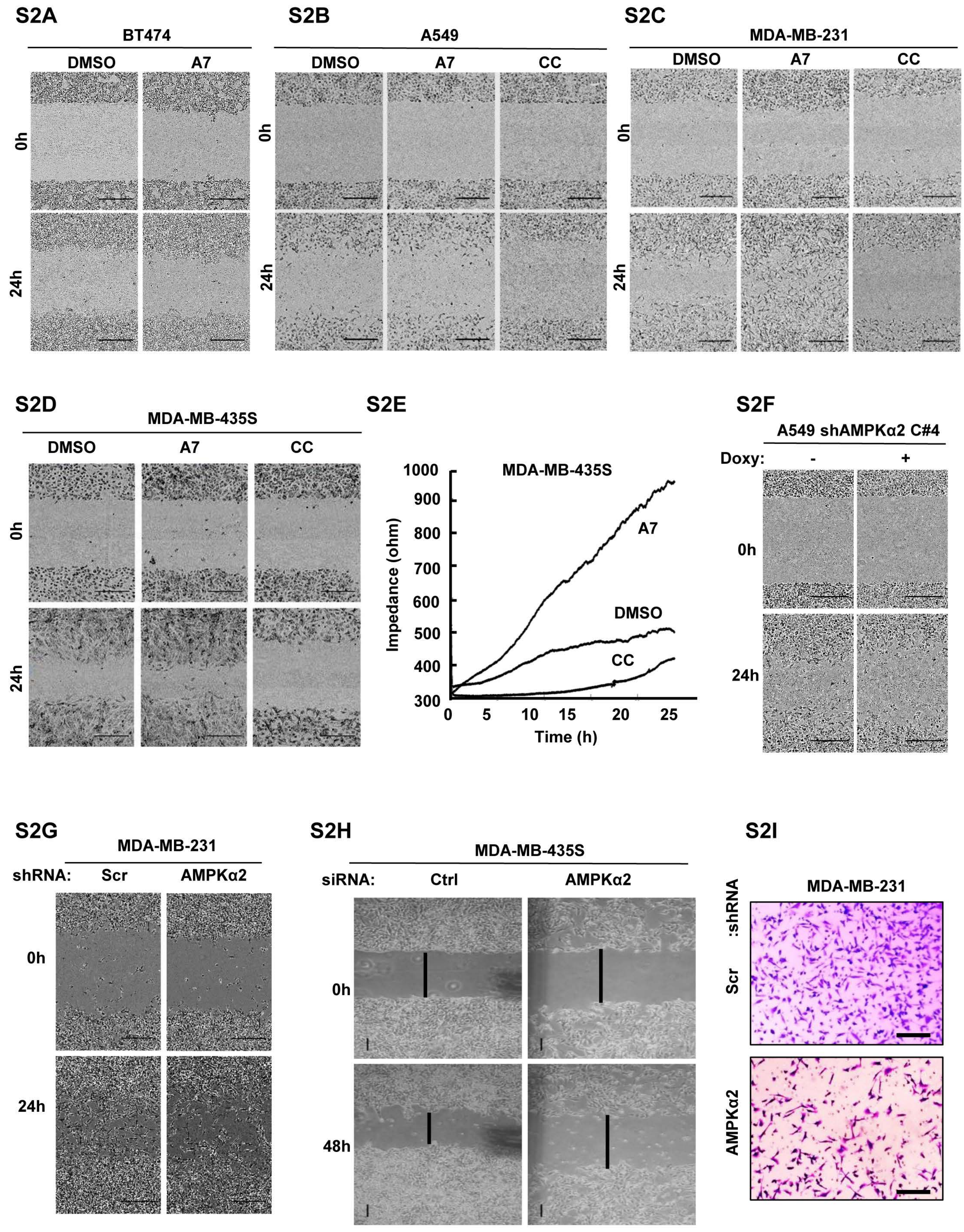
S2A
BT474
DMSO
A7
0h
24h
S2B
A549
DMSO
A7
CC
0h
24h
S2C
MDA-MB-231
DMSO
A7
CC
0h
24h
S2D
MDA-MB-435S
DMSO
A7
CC
0h
24h
S2E
MDA-MB-435S
Impedance (ohm)
1000
900
800
700
600
500
400
300
Time (h)
0
5
10
15
20
25
A7
DMSO
CC
S2F
A549 shAMPKα2 C#4
Doxy:
-
+
0h
24h
S2G
MDA-MB-231
shRNA:
Scr
AMPKα2
0h
24h
S2H
MDA-MB-435S
siRNA:
Ctrl
AMPKα2
0h
48h
S2I
MDA-MB-231
shRNA:
Scr
AMPKα2

Figure S2:

(S2A) Representative phase contrast microscopic images of the scratch assay performed with BT474 cells treated with 100 µM AMPK activator (A769662) or DMSO (vehicle control) taken between 0 and 24 hours.

(S2B) Representative phase contrast microscopic images of the scratch assay performed with A549 cells treated with 100 µM AMPK activator (A769662), 10 µM AMPK inhibitor (Compound C) or DMSO (vehicle control) taken between 0 and 24 hours.

(S2 C and D) Representative phase contrast microscopic images of the scratch assay performed with MDA-MB-231 **(C)** and MDA-MB-435S **(D)** cells treated with 100 µM AMPK activator (A769662), 10 µM AMPK inhibitor (Compound C) or DMSO (vehicle control) taken between 0 and 24 hours.

(S2E) ECIS-based migration assay was performed with MDA-MB-435S cells treated with 100 µM AMPK activator (A769662), 10 µM AMPK inhibitor (Compound C) or DMSO (vehicle control) for 24 hours. Graph shows a representative experiment of migration assessed over 24 hours from the time of wound generation; n=4.

(S2F) Representative phase contrast microscopic images of the scratch assay performed with A549 cells stably expressing inducible shRNA against AMPKα2 (clone #4) cultured with and without doxycyclin taken between 0 and 24 hours.

(S2G) Representative phase contrast microscopic images of the scratch assay performed with MDA-MB-231 cells stably expressing scrambled shRNA or AMPKα2 shRNA taken between 0 and 24 hours.

(S2H) Representative phase contrast microscopic images of the scratch assay performed with MDA-MB-435S cells transfected with control siRNA or AMPKα2 siRNA taken between 0 and 48 hours.

(S2I) Representative bright field images of the invasion assay performed with MDA-MB-231 cells stably expressing scrambled shRNA or AMPKα2 shRNA (in Fig. 2J).

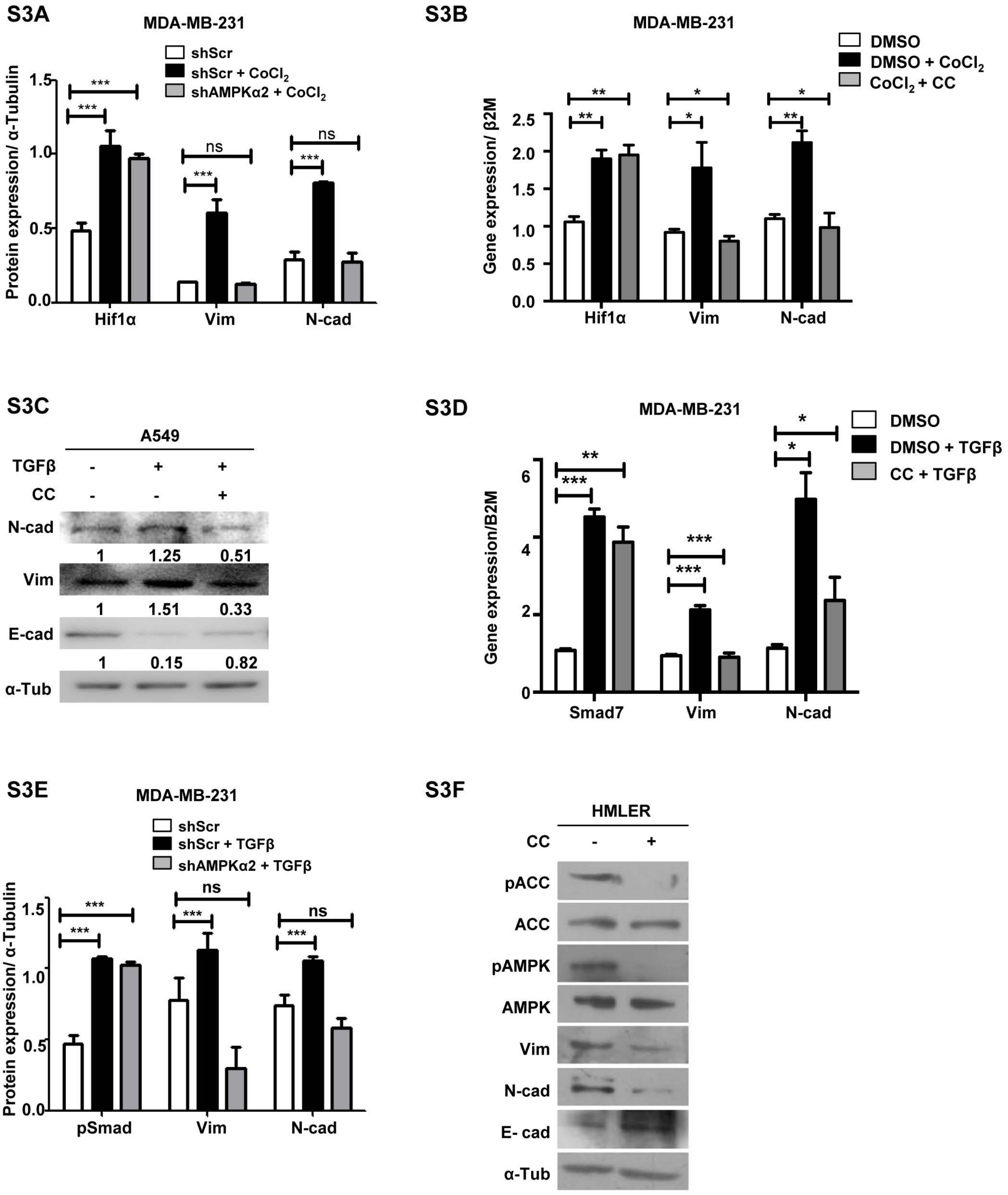

Journal of Cell Science • Supplementary information

Figure S3:

(S3A) MDA-MB-231 cells stably expressing scrambled shRNA or AMPKα2 shRNA were cultured in the presence of 150 µM $CoCl_2$ for 48 hours. Thereafter, cells were harvested and immunoblot analysis was undertaken. Graph represents densitometric quantification of the specified proteins normalized to α-Tubulin; error bars represent SEM, n=4.

(S3B) MDA-MB 231 cells were cultured with 150 µM $CoCl_2$ in the presence or absence of 10 µM AMPK inhibitor (Compound C) for 48 hours. Thereafter, cells were harvested for RNA isolation and subjected to qRT-PCR for specified transcripts. Graph represents fold change in gene expression normalized to β2m; error bars represent SEM, n=3.

(S3C) A549 cells were cultured with 5 ng/ml TGFβ in the presence or absence of 10 µM AMPK inhibitor (Compound C) for 24 hours. Thereafter, cells were harvested and immunoblot analysis was undertaken for EMT marker proteins. Band intensities are represented below the relevant panels; n=3.

(S3D) MDA-MB-231 cells were cultured with 5 ng/ml TGFβ in the presence or absence of 10 µM AMPK inhibitor (Compound C) for 24 hours. Thereafter, cells were harvested for RNA isolation and subjected to qRT-PCR for specified transcripts. Graph represents fold change in gene expression normalized to β2m; error bars represent SEM, n=3.

(S3E) MDA-MB-231 cells stably expressing scrambled shRNA or AMPKα2 shRNA were cultured in the presence of 5 ng/ml of TGFβ for 24 hours. Thereafter, the cells were harvested and subjected to immunoblot analysis. Graph represents densitometric quantification of the specified proteins normalized to α-Tubulin; error bars represent SEM, n=3.

(S3F) HMLER cells (HMLE cells overexpressing oncogenic Ras) were cultured in the presence of 10 µM AMPK inhibitor (Compound C) or DMSO (vehicle control) for 24 hours. Thereafter, cells were harvested and subjected to immunoblot analysis for the specified proteins; n=3.

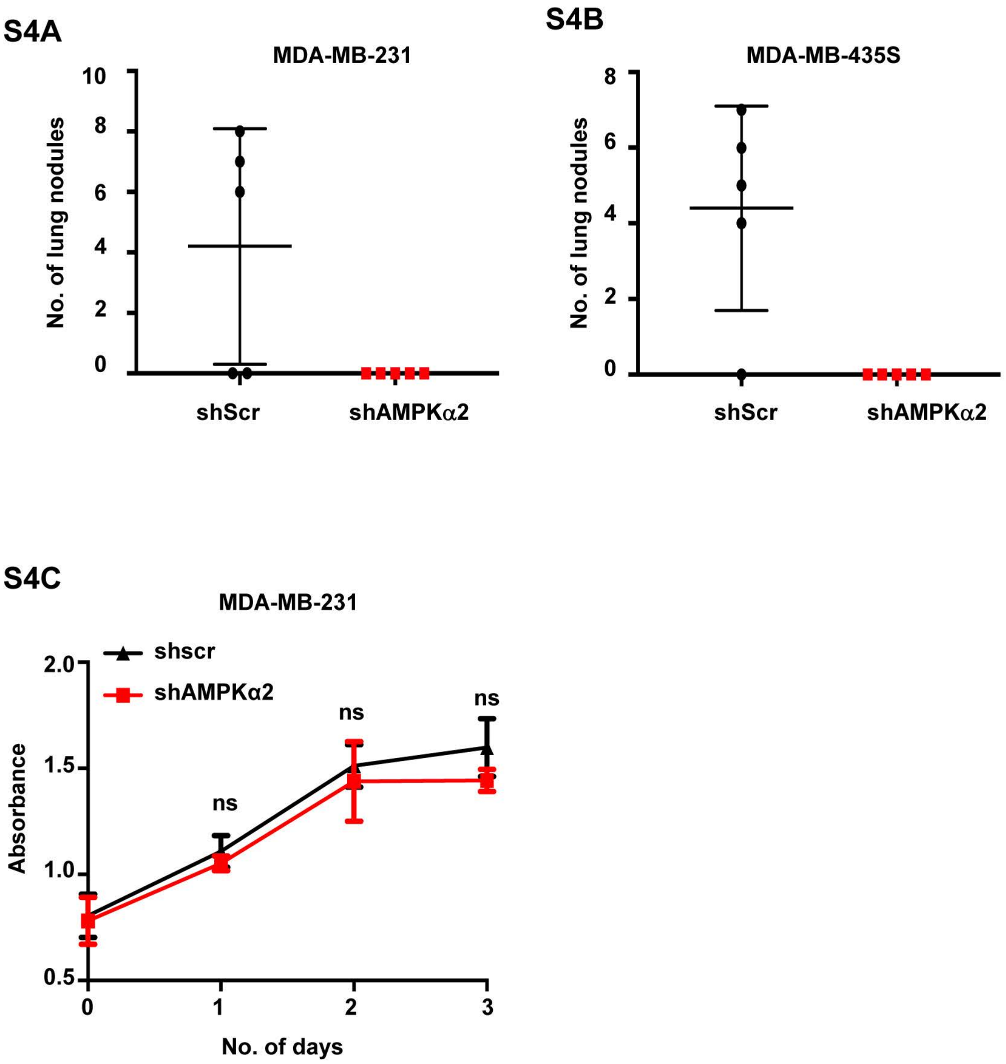

Figure S4:

(S4 A and B) Graphs represent the number of macroscopic lung nodules counted on the surface of lungs of mice injected with MDA-MB-231 **(A)** or MDA-MB-435S **(B)** cells stably expressing scrambled shRNA or AMPKα2 shRNA in Fig. 4. Each data point represents one mouse.

(S4C) Graph represents proliferation assay using MTT performed for MDA-MB-231 stably expressing scrambled shRNA or AMPKα2 shRNA; error bars represent SEM; n=3.

Journal of Cell Science • Supplementary information

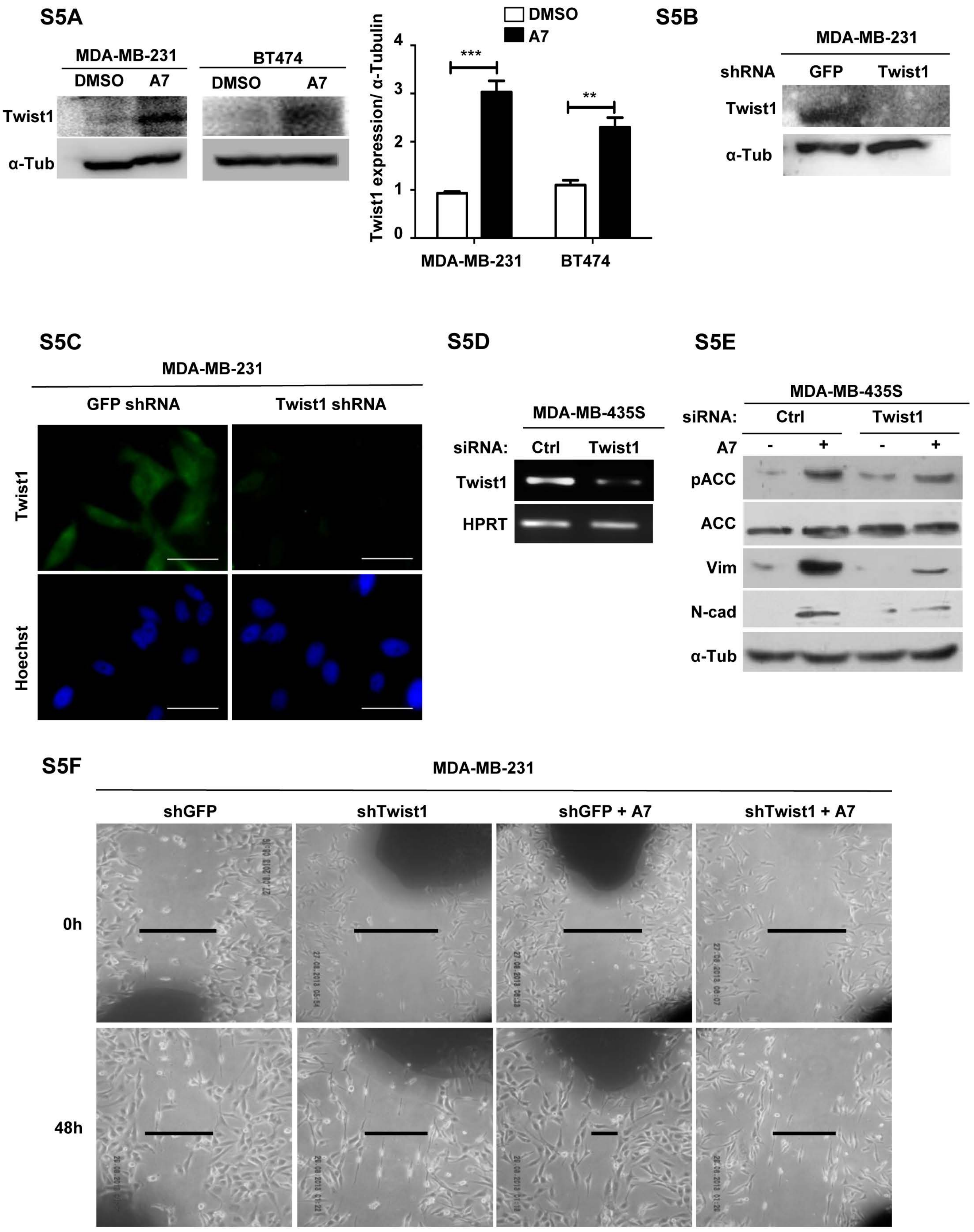
S5A
MDA-MB-231
DMSO A7
BT474
DMSO A7
Twist1
α-Tub
DMSO
A7
Twist1 expression/ α-Tubulin
***
**
MDA-MB-231
BT474
S5B
MDA-MB-231
shRNA GFP Twist1
Twist1
α-Tub
S5C
MDA-MB-231
GFP shRNA
Twist1 shRNA
Twist1
Hoechst
S5D
MDA-MB-435S
siRNA: Ctrl Twist1
Twist1
HPRT
S5E
MDA-MB-435S
siRNA: Ctrl Twist1
A7 - + - +
pACC
ACC
Vim
N-cad
α-Tub
S5F
MDA-MB-231
shGFP
shTwist1
shGFP + A7
shTwist1 + A7
0h
48h

Figure S5:

(S5A) MDA-MB-231 and BT474 cells were cultured in the presence of 100 µM AMPK activator (A769662) or DMSO (vehicle control) for 48 hours. Thereafter, cells were harvested and subjected to immunoblot analysis for Twist1 and α-Tubulin. Graph represents densitometric quantification of the Twist1 protein normalized to α-Tubulin; error bars represent SEM, n=4.

(S5B) MDA-MB-231 cells stably expressing GFP shRNA or Twist1 shRNA were subjected to immunoblot analysis for Twist1 and α-Tubulin, n=3.

(S5C) MDA-MB-231 cells stably expressing GFP shRNA or Twist1 shRNA were subjected to immunocytochemistry analysis for Twist1 protein. Photomicrograph shows representative fluorescent image taken at 20X magnification; Hoechst 33342 was used for nuclear staining, n=3.

(S5D) MDA-MB-435S cells were transfected with control siRNA or Twist1 siRNA. After 48 hours of transfection, cells were harvested and subjected to semi-quantitative PCR analysis for the specified transcripts, n=4.

(S5E) MDA-MB-435S cells transfected with control siRNA or Twist1 siRNA were cultured in the presence of 100 µM AMPK activator (A769662) or DMSO (vehicle control) for 48 hours. Thereafter, the cells were harvested and subjected to immunoblot analysis for the specified proteins; n=4.

(S5F) Photomicrographs show representative phase contrast images taken at 0 and 48 hours of scratch assay performed with MDA-MB-231 cells stably expressing GFP shRNA or Twist1 shRNA cultured in the presence of 100 µM AMPK activator (A769662) or DMSO (vehicle control) for 48 hours. Magnification, x20; n=3.

Journal of Cell Science • Supplementary information

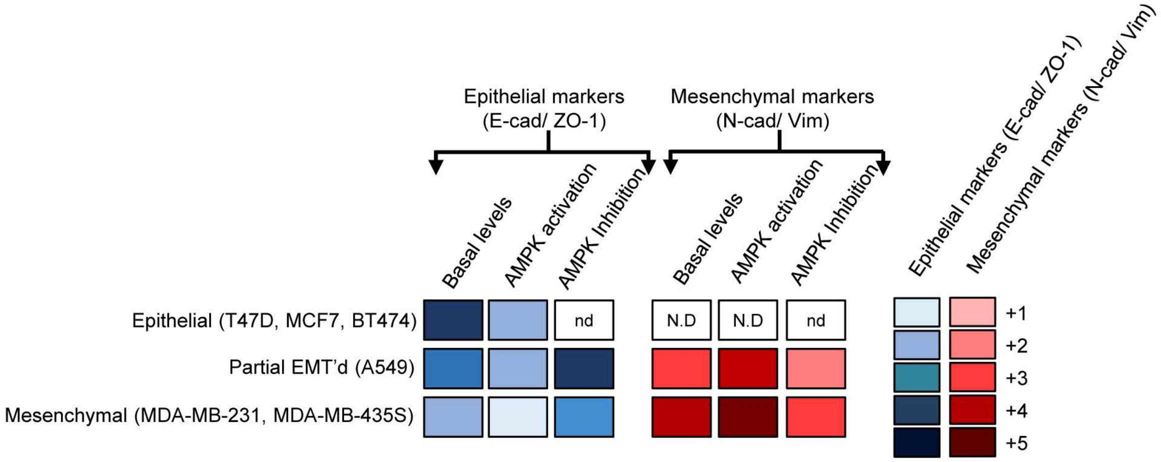

Figure S6:

(S6) A schematic representation of EMT marker expression changes at protein level upon AMPK modulation across various cancer cell lines. The basal expression level is depicted based on Fig. S1A, and alteration in protein expression is modeled based on western blot quantifications in Fig. S1B, S1D, S1F, S1G and S1H. 'N.D' signifies 'not detected' and 'nd' signifies 'not done'.

Journal of Cell Science • Supplementary information

Table S1: Primer sequences

Gene	Forward primer sequence	Reverse primer sequence
Vimentin	5'-GAGAACTTTGCCGTTGAAGC-3'	5'-TCCAGCAGCTTCCTGTAGGT-3'
N-cadherin	5'-GACAATGCCCCTCAAGTGTT-3'	5'-CCATTAAGCCGAGTGATGGT-3'
Snai1	5'-ACCCCACATCCTTCTCACTG-3'	5'-TACAAAAACCCACGCAGACA-3'
Slug	5'-CTTTTTCTTGCCCTCACTGC-3'	5'-GCTTCGGAGTGAAGAAATGC-3'
Zeb1	5'-TGCACTGAGTGTGGAAAAGC-3'	5'-TGGTGATGCTGAAAGAGACG-3'
Smad7	5'-GAATCTTACGGGAAGATCAACCC-3'	5'-CGCAGAGTCGGCTAAGGTG-3'
Hif1α	5'-GGACAAGTGACCACAGGA-3'	5'-GGAGAAAATCAAGTCGTG-3'
Twist1	5'-GTCCGCAGTCTTACGAGGAG-3'	5'-CCAGCTTGAGGGTCTGAATC-3'
β2m	5'-CCTGAATTGCTATGTGTCT-3'	5'-TGATGCTGCTTACATGTCT-3'
HPRT	5'- TGCTCGAGATGTGATGAAGG -3'	5'- TCCCCTGTTGACTGGTCATT -3'

Journal of Cell Science • Supplementary information
